# Study on secondary collapse of the bottom frame masonry structure in semi-ruined state based on FEM–FDEM

**DOI:** 10.1038/s41598-024-80612-x

**Published:** 2024-11-26

**Authors:** Defeng Xu, Feifei Sun, Huaixiang Zhao, Honghui Qi, Limei Chen, Tianyu Hu

**Affiliations:** 1 Jilin Agricultural University, Changchun, 131118 China; 2 College of Civil Engineering, Tongji University, Shanghai, 200092 China

**Keywords:** Bottom frame masonry structure, Semi-ruined state, FEM–FDEM, Aftershocks, Secondary collapse mechanisms, Response characteristics, Civil engineering, Natural hazards

## Abstract

The bottom frame masonry structure (BFMS) in a semi-ruined state is vulnerable to secondary collapse under strong aftershocks, posing a significant risk to rescuers during post-earthquake operations. Therefore, investigating the mechanisms and characteristics of secondary collapse of BFMS in a semi-ruined state (BFMS-SR) under aftershocks is critical. This paper proposes a numerical modeling framework for BFMS under mainshock-aftershock conditions, utilizing a combination of the finite element method (FEM) and the finite discrete element method (FDEM). The validation demonstrates that FEM–FDEM can effectively reproduce the transition of BFMS from an intact state to a semi-ruined state, ultimately leading to a secondary collapse state. Subsequently, the mechanisms and structural response characteristics of BFMS-SR under aftershocks are analyzed. The secondary collapse of the BFMS-SR under aftershocks is primarily governed by column hinge development. Furthermore, according to the time-history curve of absolute vertical velocity, the secondary collapse of BFMS-SR exhibits four distinct stages: stabilization, structural response development, secondary collapse, and post-collapse. The end of the stabilization phase is proposed as the early-warning threshold for secondary collapse of BFMS-SR under aftershocks, aiding in post-earthquake rescue operations.

## Introduction

**Fig. 1:**
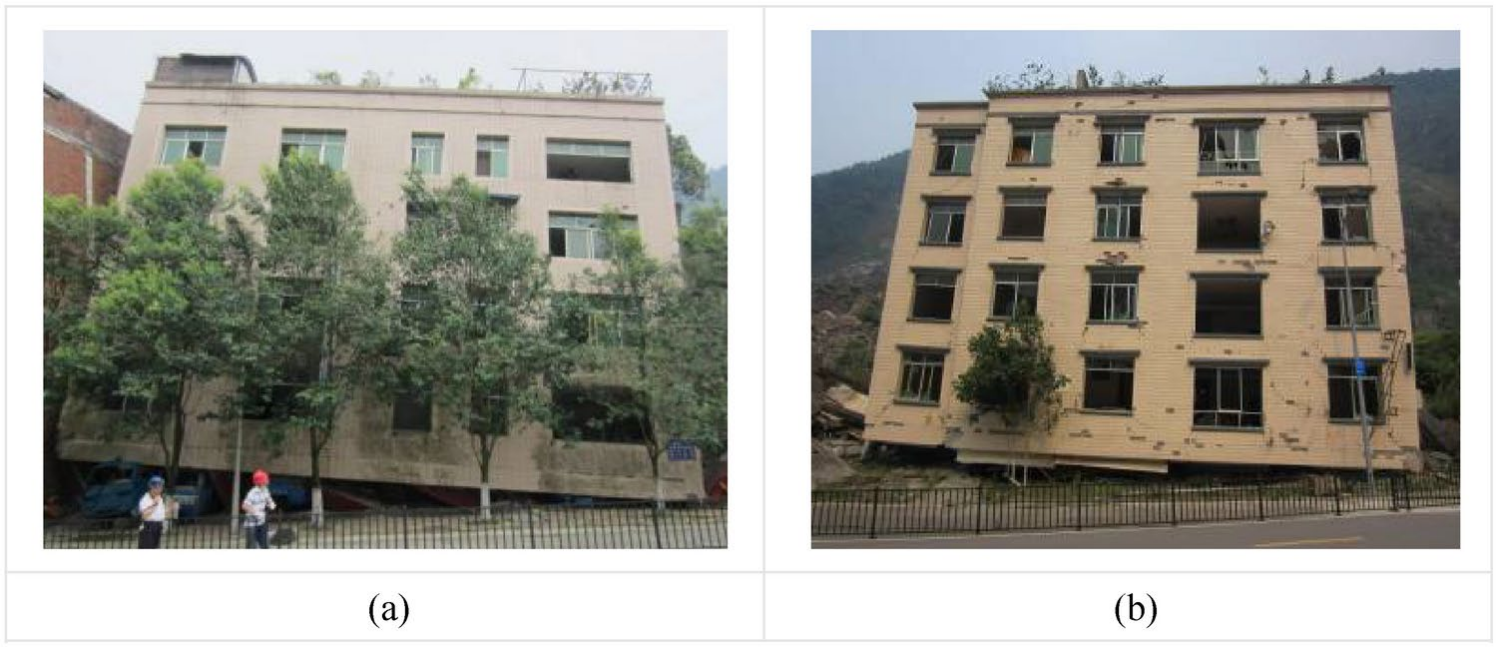
Collapse state of BFMS in Beichuan County, Wenchuan.

The bottom frame masonry structure (BFMS) is a mixed load-bearing system comprising a reinforced concrete frame (RCF) at its base and a masonry structure in the upper part. BFMS is vulnerable to transitioning into a semi-ruined state characterized by survival spaces at the bottom under rare earthquakes^1,2^. Notably, approximately 80% of BFMS collapsed into a tilting semi-ruined state during the 2008 Wenchuan Ms 8.0 earthquake in China (see Fig. 1)^3–5^. Furthermore, BFMS in a semi-ruins state (BFMS-SR) are susceptible to secondary collapse under strong aftershocks. The secondary collapse of building ruins caused by strong aftershocks threatens the safety of both trapped individuals and rescuers. For instance, more than 30 rescuers and volunteers lost their life due to the secondary collapse of building ruins during the rescue operations in the 2008 Wenchuan earthquake^6^. Therefore, to safeguard rescue operations, it is imperative to conduct research on the mechanisms and structural response characteristics of secondary collapse in BFMS-SR under aftershocks.

Research on secondary collapse of building ruins, including BFMS-SR, primarily focuses on post-earthquake safety rescue issues. The related research encompass rapid assessment, rescue early-warning, and simulation methods for secondary collapse of building ruins under aftershocks. The rapid assessment of building ruins is crucial in safeguarding rescuers and enhancing rescue efficiency. Researchers have introduced rapid assessment methods for various types of building ruins (including RCF structures and prefabricated masonry structures) through investigations of simplified mechanical models of semi-ruined structures. Simultaneously, the relationship between critical collapse PGA (Peak Ground Acceleration) and the tilt angle of building ruins has been established, providing a theoretical foundation for safety assessors to conduct rapid assessments^7,8^. However, the aforementioned research overlooks the crucial role of collapse mechanisms in simplifying mechanical models of building ruins.

The rescue early-warning method primarily focuses on monitoring the structural dynamic response changes of building ruins and shoring components under aftershocks. For instance, total stations are employed to monitor the displacement and tilt angle parameters of the building ruins, facilitating on-site early-warning (Federal Emergency Management Agency^9^. Additionally, sensors installed in wood shoring^10^ can trigger alerts when pressure and deformation thresholds are exceeded. However, the above-mentioned early-warning methods confront challenges in swiftly detecting risks and delivering precise predictions. A direct early-warning method need be developed through accurately capturing the structural response characteristics of secondary collapse in building ruins under aftershocks. FeiFei Sun^11^ proposed a dynamic post-earthquake early-warning system for secondary collapse of building ruins under aftershocks. Nevertheless, the characteristics of secondary collapse in building ruins, including BFMS-SR, has rarely investigated in prior research. To address the issues related to mechanisms and characteristics analysis, both shaking table tests and numerical simulations are effective approaches.

In reality, the numerical simulation methods related to secondary collapse should consider three factors: the influence of infilled walls on the seismic collapse of overall BFMS; the connections between infilled walls and bottom RCF structure, as well as between structural columns (ring beams) and masonry walls; and maximizing the retention of essential BFMS model elements. Researchers have employed various methods to simulate seismic collapse of BFMS for RCF and masonry structures, including the Finite Element Method (FEM)^5,12,13^, Extended Discrete Element Method (EDEM)^2,14^, Finite Discrete Element Method (FDEM)^15–17^, and Applied Element Method (AEM)^18,19^. FEM achieves the seismic collapse of BFMS at the cost of “killing” a certain number of BFMS model elements, which limits the retention of model elements. EDEM and FDEM are commonly employed to simulate infilled wall and masonry wall structures. Nevertheless, while they are advantageous in simulating infilled walls and masonry walls, simulating RCF poses greater challenges. AEM can be utilized to establish BFMS models based on Extreme Loading for Structures (ELS) program. However, the ELS program currently does not provide a continuous calculation function. Additionally, determining the failure criteria for the spring element is challenging, which affects the final collapse mode of BFMS to some extent. Since the aforementioned numerical simulation methods focus on the anti-collapse mechanisms of BFMS under mainshocks, studies on safety rescue early-warning under aftershocks are limited.

It is noteworthy that research on the mechanisms and structural response characteristics related to secondary collapse in BFMS-SR under aftershocks faces several challenges. Firstly, there is a notable absence of an effective and accurate numerical simulation method for the seismic collapse of BFMS under mainshock-aftershock scenarios that considers the three aforementioned rescue-related factors. Furthermore, there is currently a lack of selection principles for aftershock sequences and discrimination methods for identifying secondary collapses of BFMS-SR.

**Fig. 2:**
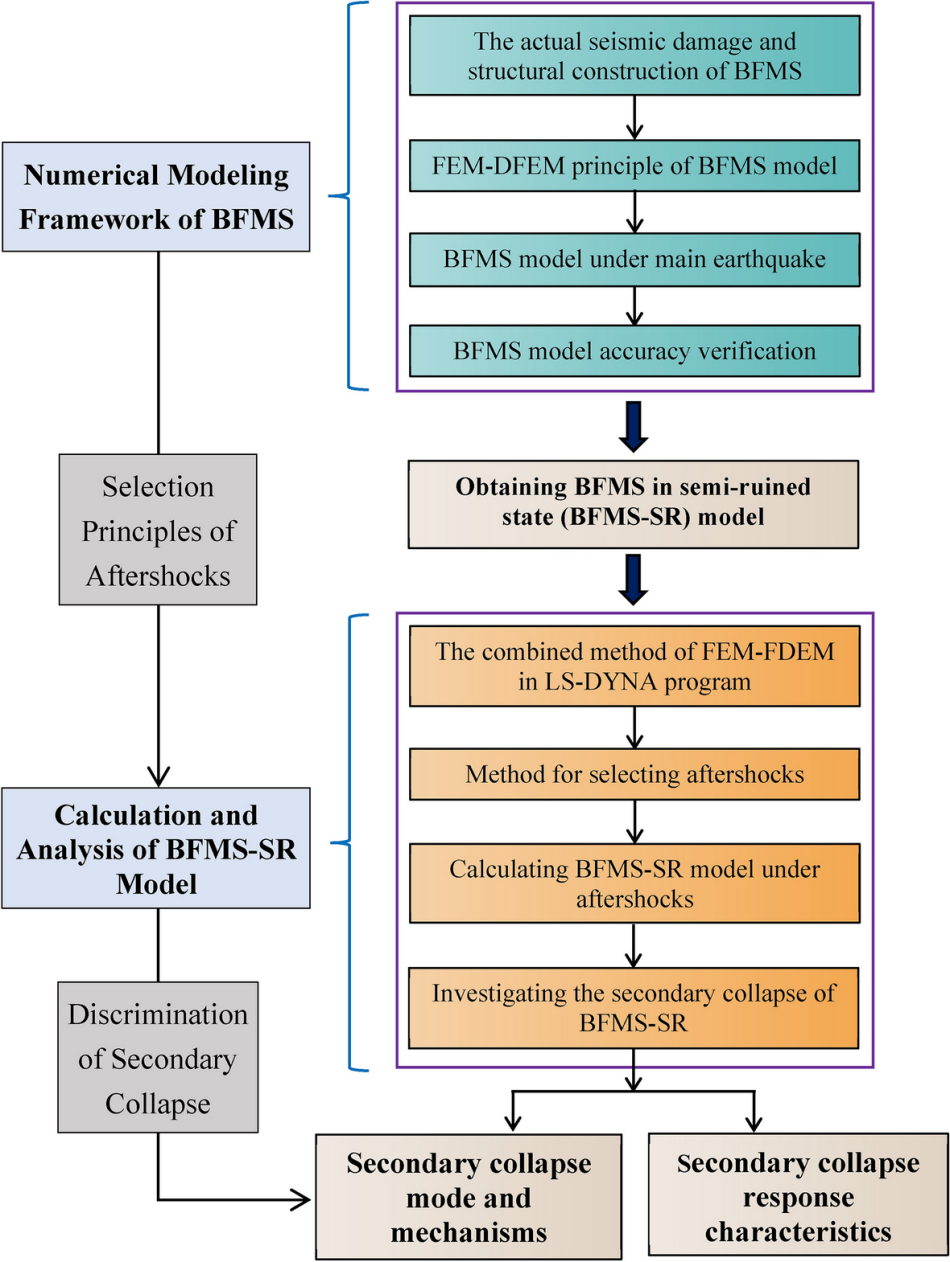
The research content flowchart.

This study addresses the challenge of accurately simulating the collapse of BFMS (comprising RCF structure elements, infill wall elements, masonry wall elements, and their interconnection elements) under mainshocks and aftershocks. Section “Numerical modeling framework of BFMS” introduces a numerical modeling framework of BFMS based on FEM–FDEM in the LS-DYNA program. Section “Numerical simulation case” examines the numerical simulation of a BFMS case under mainshock-aftershock scenarios and verifies the accuracy of the BFMS model. Based on the validated numerical model, the modes and mechanisms of secondary collapse for the BFMS-SR model are explored in Section “Mode and mechanism of secondary collapse for BFMS-SR”. Finally, the structural response characteristics of secondary collapse in BFMS-SR under aftershocks are investigated in Section “Main characteristics of secondary collapse of BFMS-SR model”. A flowchart is included to provide a clear overview of the paper’s content, as illustrated in Fig. 2.

## Numerical modeling framework of BFMS

Existing research has demonstrated that FEM can accurately replicate the spalling of concrete and fracture of reinforcement bars in RCF beam and column components. FDEM can effectively simulate the progression of infilled and masonry walls from the initial to collapse state^8,19,17^. Notably, prior research by Defeng Xu^8^ has revealed that the combination of FEM and FDEM within the LS-DYNA program can effectively leverage the respective advantages of these two approaches. Therefore, the FEM–FDEM framework is expected to simulate BFMS from the intact state to the semi-ruined and secondary collapse states under both mainshock and aftershock scenarios accurately and scientifically.

**Fig. 3:**
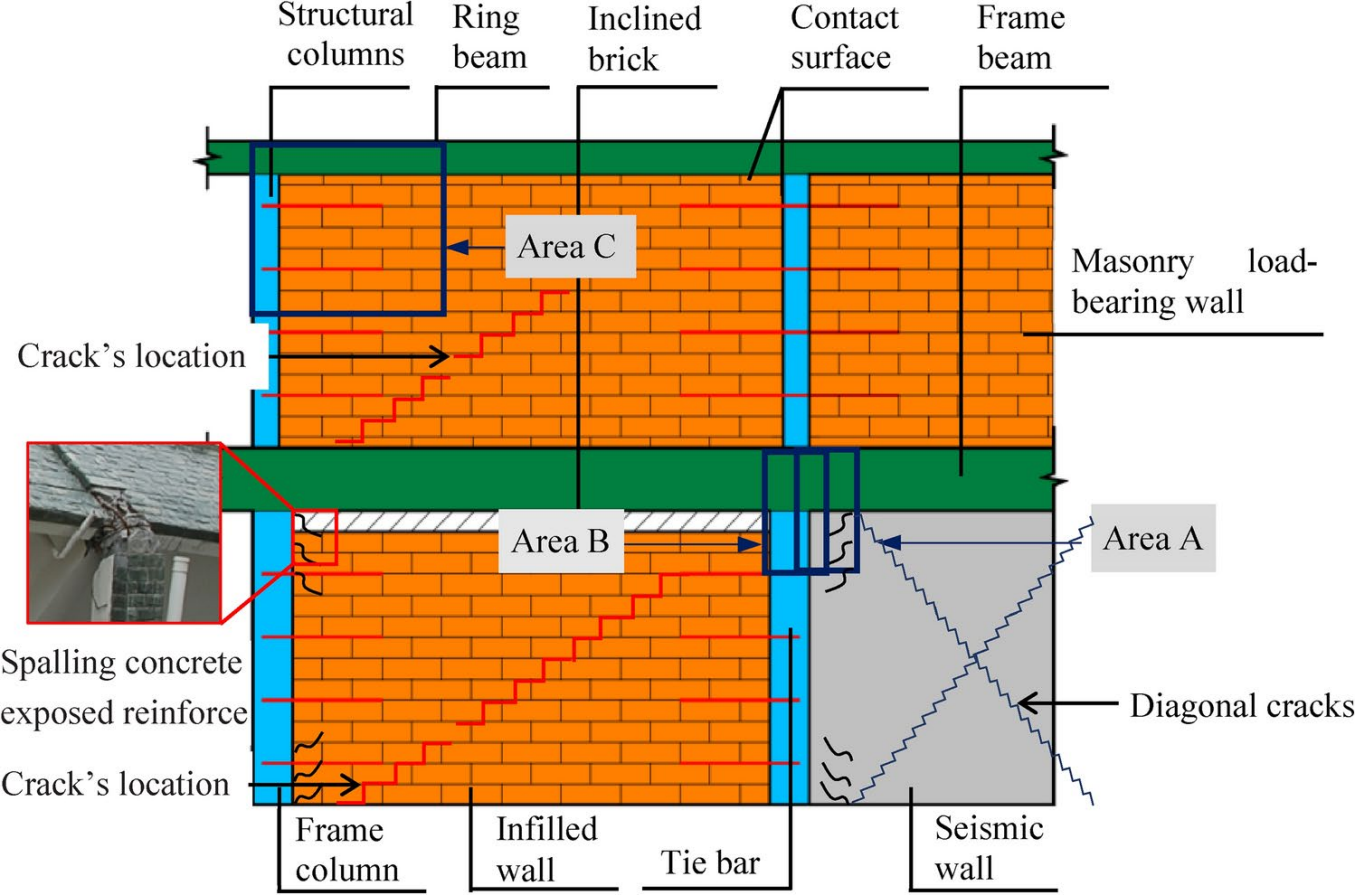
The structural construction and seismic damage schematic diagram of BFMS.

According to the “Code for Design of Masonry Structures”^20^, tie reinforcement bars are employed to connect the infill wall to the bottom RCF columns and the masonry wall to the structural column. Additionally, top inclined brick construction measures are typically implemented between the infill wall and the top RCF beam to prevent the transmission of vertical loads through a “soft connection.” The contact surface between the upper masonry load-bearing wall and the structural columns and ring beams consists of mortar and tie reinforcement bars, respectively. Referring to the “Code for Seismic Design of Buildings” (China Construction Industry^21^, seismic walls are required in the bottom RCF structure. Based on actual earthquake damage investigations^22,23^ and earthquake simulation shaking table tests^1,24^, the failure phenomenon of the bottom RCF is characterized by concrete crushing and peeling at the column ends, yielding of the steel bars, tilting of the bottom layer, ultimately leading to overall structural instability and collapse. The failure of the infilled wall in the bottom layer is evidenced by the initiation, propagation, and collapse of cracks on the masonry block contact surfaces. The failure phenomenon of the bottom seismic wall is indicated by the development of cross-diagonal cracks and collapse alongside the RCF columns. The failure of the upper masonry structure is evidenced by numerous cracks and partial collapse at the block contact surfaces. The structural construction and seismic damage schematic of BFMS is presented in Fig. 3.

**Fig. 4:**
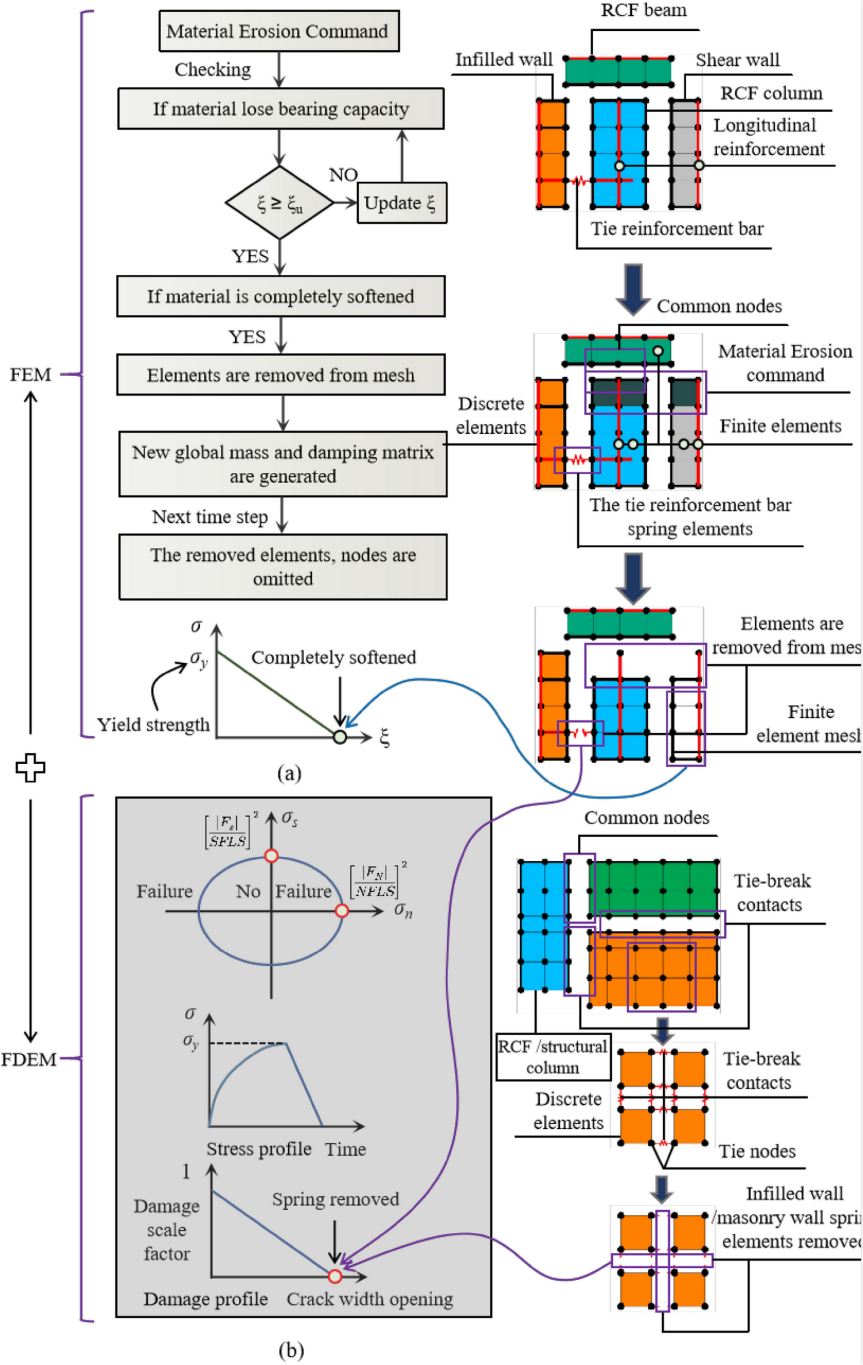
The modeling framework of FEM–FDEM in BFMS model.

According to the actual structural construction and seismic damage characteristics of the BFMS, a numerical simulation framework based on FEM–FDEM for the seismic collapse of BFMS has been proposed. This framework employs the Element Deletion Technique (EDT) in FEM to simulate various components, including the bottom RCF beams, slabs, and columns, as well as the upper RC structural columns and ring beams. The EDT principle involves removing finite elements from the finite element meshes when the strength vanishes during the strain-softening process. A single time step of the algorithm is typically divided into multiple phases, as illustrated in Fig. 4a. Area A in Fig. 3 is selected as the research object, where the concrete and reinforcement bars of the RCF beams, columns, and shear walls are modeled using the separation method. Subsequently, finite element meshes are divided, and common nodes are processed. When the material strain in the column, beam, shear wall, and reinforcement bar elements exceeds the limit strain, the corresponding elements are deleted. However, it’s essential to note that EDT has certain drawbacks, such as its high dependence on mesh size and the non-conservation of mass and momentum in the deleted regions, particularly in large-scale element models^25^. Thus, it is crucial to ensure that finite element sizes are appropriately small.

The Node Release Technology (NST) in FDEM is employed to simulate both infill and masonry walls. NST is also used to model the connections between the masonry wall and the RC structural column (ring beam), as well as the connections between the infill wall and the RCF column (beam). NST was initially developed and applied by Vignjevic et al.^26^ and Bala^27^. In NST, tie-break contacts are established between elements when the wall blocks are discretized with a finite element mesh. A pair consisting of a slave node and a master segment constitutes the basic unit of tie-break contacts. A slave nodal force is calculated in proportion to the penetration depth, and an internally computed contact stiffness is utilized. The master segment, located between the slave node and the contact point, acts as a spring, transmitting both normal and shear forces, and includes optional failure criteria. Failure is assumed when the squared normalized radius of the circle reaches or exceeds 1, leading to the deactivation of the spring, as illustrated in Eq. (1) and Fig. 4b. Damage initiation occurs once the yield stress is reached. After damage initiation, the stress is linearly reduced until the crack width reaches a specified value. At that point, the spring stress decreases linearly to zero, and the spring is removed^27^.

1 $$\left[ {\frac{{\left| {F_{N} } \right|}}{NFLS}} \right]^{2} = \left[ {\frac{{\left| {F_{s} } \right|}}{SFLS}} \right]^{2} \ge 1$$

where *NFLS* is normal failure force or stress, *SFLS* is shear failure force or stress, *F*_*N*_ is spring normal force or stress, and *F*_*S*_ is spring shear force or stress.

Areas B and C (as indicated in Fig. 3) were chosen as the focus of this study. To ensure the in-plane and out-plane seismic performance of both the infill wall and the masonry wall, tie reinforcement bars were installed between the infill wall and the RCF column, as well as between the masonry wall and the structural column, in accordance with the seismic design code of buildings in China (China Construction Industry^21^. The interaction between the infill wall and the bottom RCF beam is primarily based on their contact interaction post-deformation. Consequently, tie-break contacts in NRT were established on the contact surface between the infill wall and the bottom RCF column (beam), and between the masonry wall and the structural column (ring beam), simplifying the previously mentioned tie-break spring.

**Fig. 5:**
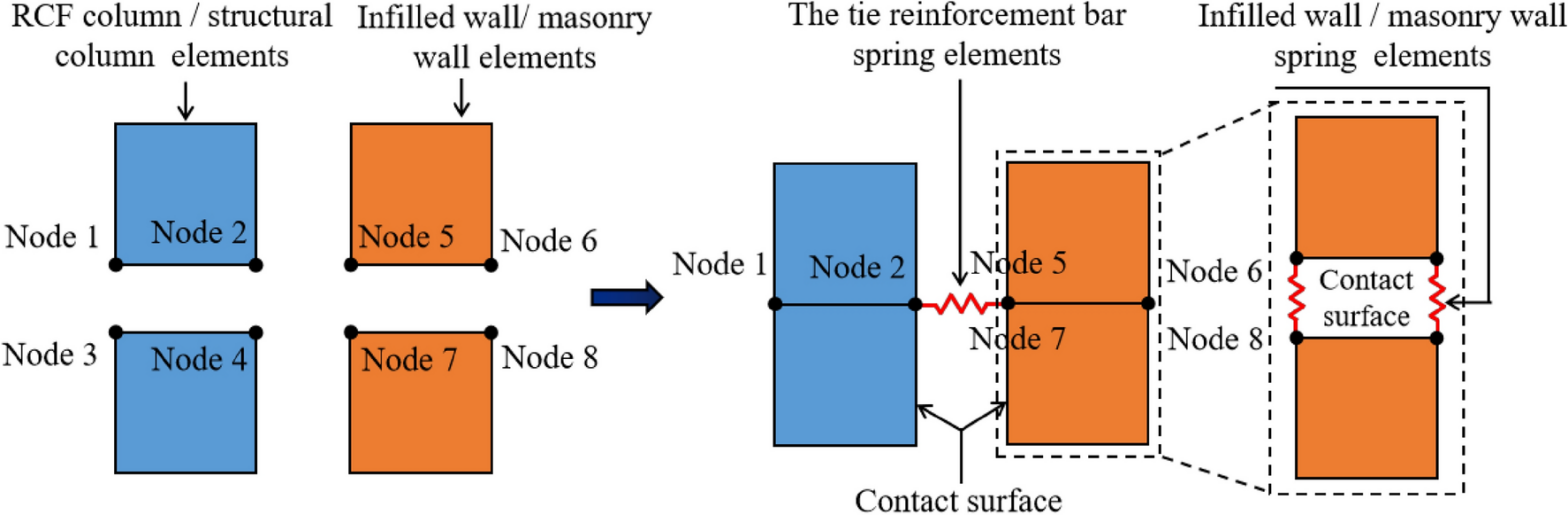
The relationship between infilled wall infilled wall (masonry wall) and RCF column (structural column) elements in FEM–FDEM model.

To establish the structural connection between the infill wall and the RCF column in the BFMS numerical model, the RCF column element mesh shared common nodes, where node 1 was merged with node 3, and similarly, node 2 with node 4. However, the same coordinate nodes of the infill wall elements were not merged. Instead, springs were set between nodes following the principles of tie-break contacts in NST. The tie reinforcement bar between the RCF column and the infill wall was modeled as springs, which were set between nodes 2, 5, and 7, as illustrated in Fig. 5. The calculation of the equivalent spring for the tie reinforcement bar is detailed in Eq. (2).

2 $$F_{cy} = \frac{{m \times F_{y} }}{n}$$

where *F*_*cy*_ represents the stress in the equivalent spring of the tie reinforcement bar, *m* denotes the number of tie bars between the infilled wall and the bottom RCF column, *F*_*y*_ indicates the tensile or shear strength of the tie reinforcement bars, *n* represents the number of springs between the infilled wall and the bottom RCF column in the BFMS numerical model. *F*_*cy*_ corresponds to NFLS when *σ*_*cy*_ represents the tensile force or stress in the tie reinforcement bars, and to SFLS when *F*_*y*_ represents the shear force or stress in the tie reinforcement bars.

**Fig. 6:**
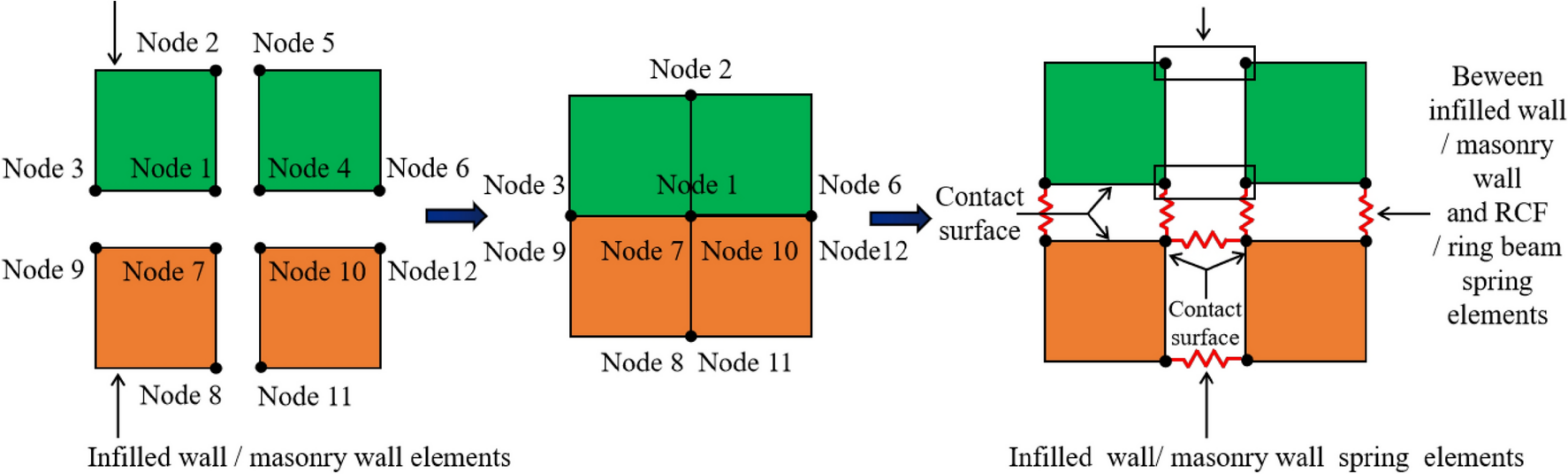
The relationship between infilled wall (masonry wall) and RCF (ring beam) elements in FEM–FDEM model.

Figure 6 depicts the structural relationship between the infilled wall and the RCF beam within the numerical model. The common node method was applied, merging node 1 with node 4 and node 2 with node 5. However, the same coordinate nodes of the infilled wall elements were not merged. Instead, springs were introduced between the nodes of the infilled wall elements in accordance with the principles of Tie-break contacts in NRT. Analogous springs were established between the nodes of the infilled wall and the RCF beam elements. The *NFLS* and *SFLS* values were set to 0, creating a “soft connection” between the RCF beam and the infilled wall. The relationships between the masonry wall and the ring beam, as well as the structural column, are established in a similar manner.

## Numerical simulation case

### Numerical model of BFMS

**Fig. 7:**
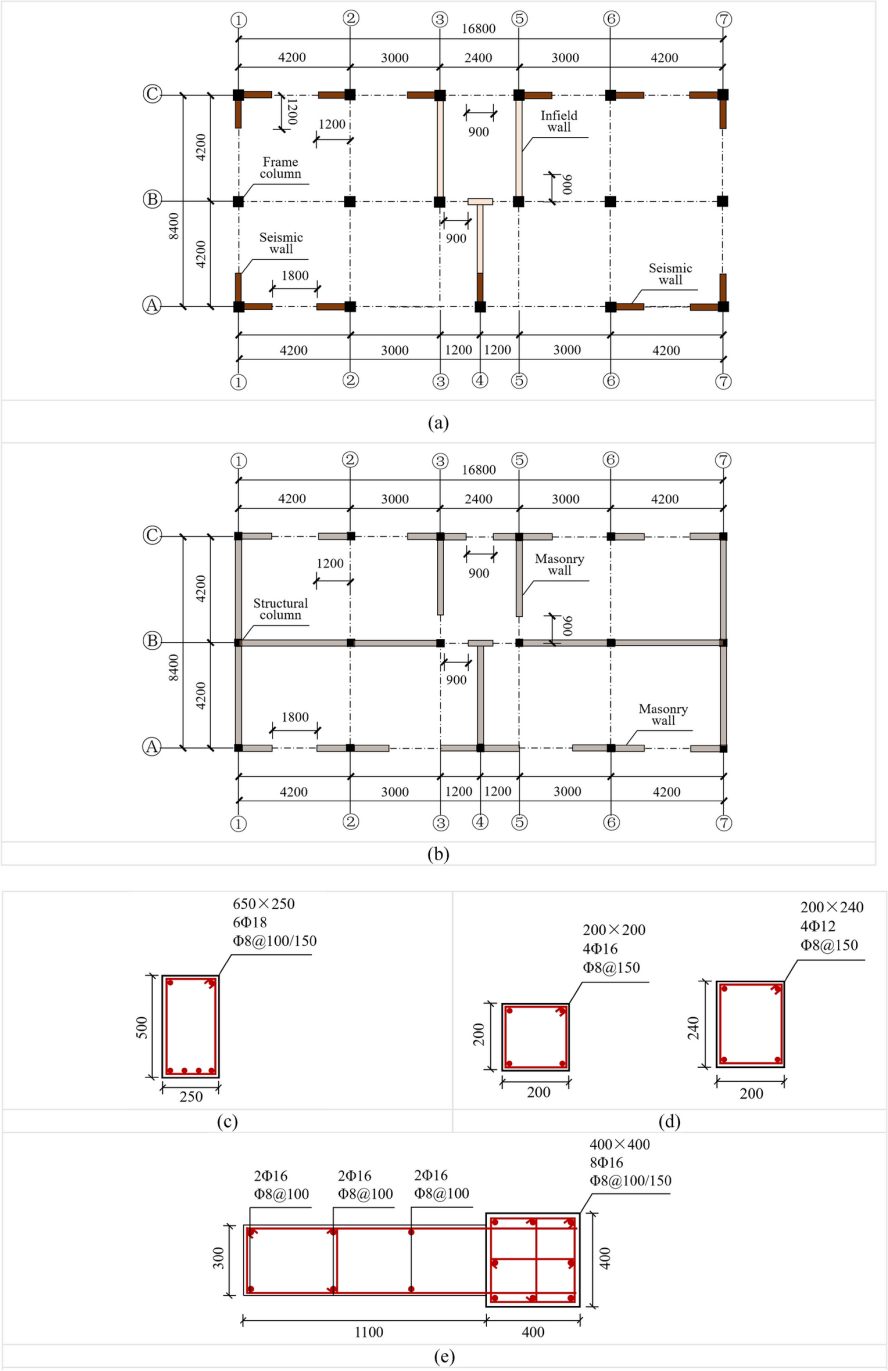
Architectural drawing and reinforcement drawing of BFMS (unit: mm): (**a**) First floor architectural plan drawing. (**b**) Second to fifth floor architectural plan drawing. (**c**) Frame column and beam reinforcement drawing. (**d**) Structural column and ring beam reinforcement drawing. (**e**) Seismic wall reinforcement drawing.

The numerical model is based on a collapsed BFMS from the 2008 Wenchuan M8.0 earthquake in China. The earthquake is classified as a first-group event, with a seismic precautionary intensity of 7 and a site category of II. The bottom RCF column has a cross-sectional size of 400 mm × 400 mm, the RCF beam has a cross-sectional size of 200 mm × 500 mm, with a floor thickness of 100 mm, a masonry wall thickness of 200 mm, and a structural column cross-sectional size of 200 mm × 200 mm. The ring beam has a cross-sectional size of 200 mm × 200 mm. The height of the first layer is 4500 mm, while the heights of the second through fourth layers are 3500 mm. The material strength grades of the concrete, reinforcement bars, blocks, and mortar are C30, HRB335, Mu10, and Mb10, respectively. The architectural and structural reinforcement drawings are presented in Figs. 7.

**Fig. 8:**
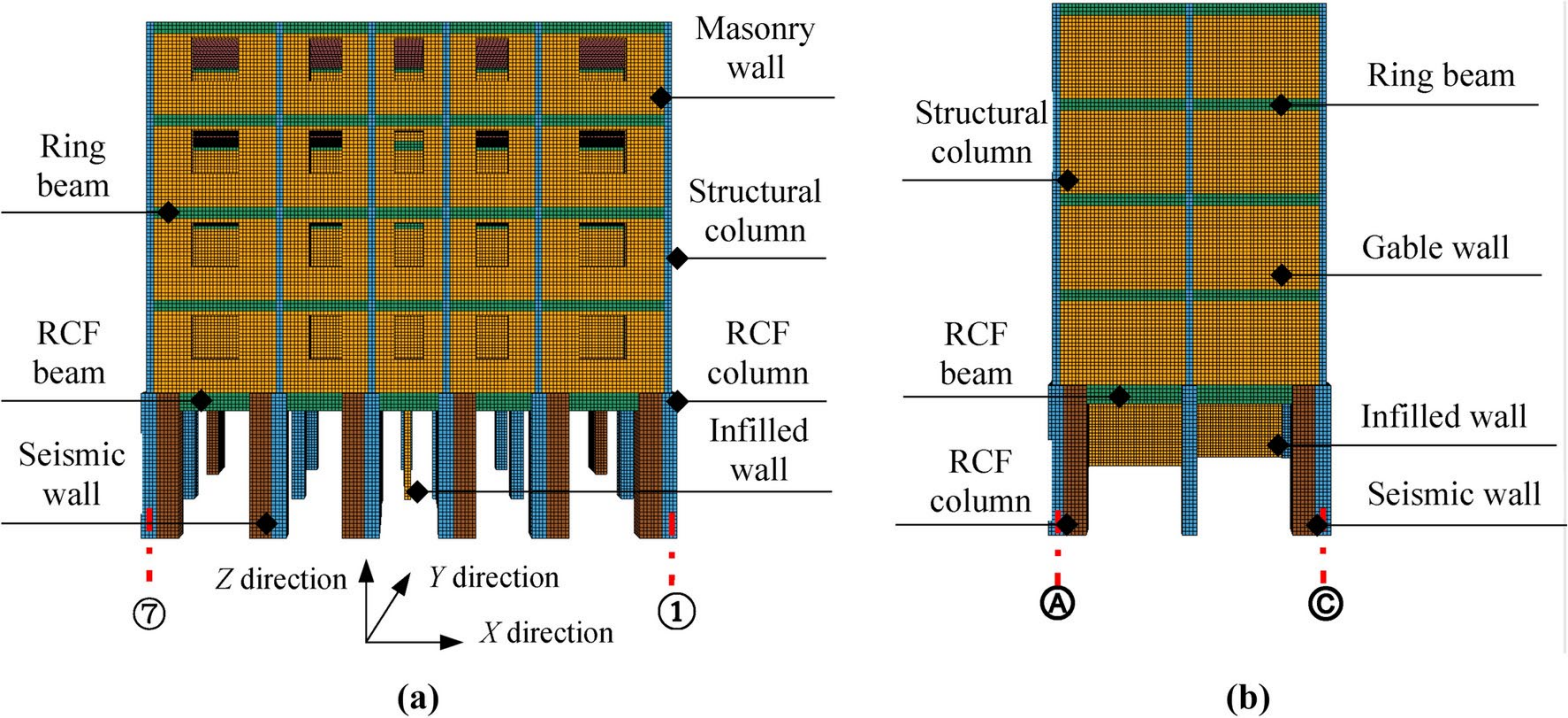
⑦-①-axle the numerical model of the BFMS. Ⓐ-Ⓒ-axle the numerical model of the BFMS.

Figure 8 shows the numerical model of the BFMS. Concrete is simulated using 8-node solid elements with reduced integration. To mitigate the hourglass effect, hourglass control type 5 and a coefficient of 0.05 are used. The reinforcing bars are modeled using 2-node Hughes-Liu beam elements with 2 × 2 Gauss quadrature integration at the cross-section. The BFMS model comprises 49,670 beam elements and 247,266 solid elements.

### Materials

The Karagozian & Case (K&C) Model (Mat_72REL3 in LS-DYNA R10.0)^28^ is used to simulate the concrete material. The concrete material parameters are presented in Table 1. The Cowper-Symonds model (Mat_003 in LS-DYNA R10.0)^29^ is used to simulate the reinforcement bar material. This model is capable of capturing isotropic and kinematic hardening plasticity as well as rate effects. The key parameters of the model include Young’s modulus, yield stress, tangent modulus, ultimate strain, and strain rate. In mechanical property tests conducted by Lin et al., the ultimate tensile strain for HRB335 was determined to be 0.015^30^. The material parameters for the reinforcement bars are presented in Table 2. The Winfrith-Concrete model (Mat_084 in LS-DYNA R10.0)^31^ is used to simulate the material of the infilled wall and masonry wall. This model is characterized by smeared crack behavior and features a smeared rebar model implemented in the 8-node single integration point continuum element. The key parameters for this model include density, Young’s modulus, uni-axial compressive strength, uni-axial tensile strength, and crack width. The material parameters for the infilled wall and masonry wall are provided in Table 3.

**Table 1:** Cowper-Symonds model parameters (unit: *N*, *m*, *Pa*, *s*).

Uniaxial tensile strength (*f*_*t*_)	Density (*ρ*)	Unit conversion parameters		Ultimate tensile strain (*ξ*_*u*_)
1.43E+06 Pa	2.5E+03 kg/m^3^	39.37	1.45E−4	0.021

**Table 2:** Cowper-symonds model parameters (unit: *N*, *m*, *Pa*, *s*).

Density (*ρ*)	Elastic modulus (*E*)	Yield strength (*σ*_*y*_)	Tangent modulus (*E*_*t*_)	Ultimate tensile strain (*ξ*_*u*_)
7.8E+03 kg/m^3^	2.0E+10 Pa	3.35E+08 Pa	2.0E+09 Pa	0.011

**Table 3:** WINFRITH-CONCRETE model parameters (unit: *N*, *m*, *Pa*, *s*).

Density (*ρ*)	Elastic modulus (*E*)	Uni-axial compressive strength (*f*_*c*_)	Uni-axial tensile strength (*f*_*t*_)	Ultimate crack width *W*_*c*_
1.8E+03 kg/m^3^	2.05E+10 Pa	1.89E+07 Pa	1.89E+06 Pa	0.0024 m

The parameters refer to the “Code for Design of Masonry Structures (GB50003-2011)” and the “Code for Seismic Design of Buildings (GB50011-2010)” in China.

### Contacts

Tie-Break contact in LS-DYNA is used to simulate the relationships among the infilled wall, RCF column, and RCF beam, as well as the infilled wall itself. The Tie-Break contact model includes both non-automatic and automatic options^27^. The automatic Tie-Break contact with stress-based failure was used due to the ease of determining the material failure stresses of the tie reinforcement bars and the mortar between the bricks of the infilled wall. Thus, *F*_*N*_ and *F*_*S*_ in formula (1) represent the normal and shear stresses of the spring, respectively. Specifically, CONTACT_AUTOMATIC_SURFACE_TO_SURFACE_TIEBREAK and damage modeling (OPTION = 6) were selected, as illustrated in Fig. 4b (Tables 4 and 5).

Figure 4a illustrates the modeling scheme for the connections between the infilled wall, RCF column, and RCF beam. The failure limit for equivalent normal stress (*NFLS*) is calculated as *NFLS* = *N* × *σ*_*y*_/*n* = 5.204 × 10^6^ Pa, where *N* represents the number of tie bars (136), *σ*_*y*_ is the tensile strength of the tie reinforcement bar (3.35 × 10^8^ Pa from testing), and *n* is the number of normal springs (8754). Similarly, according to the code for design of concrete structures in China (China Construction Industry^32^, the shear strength of reinforcement bar can be approximately regarded as equal to the tensile strength. Therefore, the failure limit of equivalent shear stress, *SFLS,* is calculated as *SFLS* = 5.204 × 10^6^ Pa. The friction coefficient (FS/FD) between infilled wall and RCF column (beam) is set to 0.7^33^. Main parameters for LS-DYNA keywords are listed in Table 4. Furthermore, based on the code for the design of masonry structures in China^20^, the mortar shear failure stress *SFLS* = *k*_5_ (*f*_2_)^1/2^, where *k*_5_ is 0.125 and *f*_2_ is the average compressive strength of mortar (9.8 × 10^6^ pa from testing). The tensile strength is approximately regarded as equal to shear strength. Consequently, the mortar normal and shear failure stress are *SFLS* = *NFLS* = 0.39 × 10^6^ pa. Similarly, the mortar normal and shear failure stress of masonry wall are *SFLS* = *NFLS* = 0.412 × 10^6^ pa. The friction coefficient (FS/FD) of blocks between infilled wall and masonry wall is set to 0.7^33^. Main parameters of keywords in LS-DYNA is shown in Table 4. The contact model for overall BFMS adopts *CONTACT_ERODING_SINGLE_SURFACE to prevent the mutual penetration issues among solid elements.

**Table 4:** Main parameters of *CONTACT_AUTOMATIC_SURFACE_TO_SURFACE_TIEBREAK.

Parameters	FS	FD	OPTION	*NFLS* (Pa)	*SFLS* (Pa)
Between RCF and infilled wall	0.7	0.7	6	5.204 × 10^6^	5.204 × 10^6^
Infilled wall	0.7	0.7	6	0.39 × 10^6^	0.39 × 10^6^
Masonry wall	0.7	0.7	6	0.421 × 10^6^	0.421 × 10^6^

### Mainshock loading cases

Referring to the loading conditions used in the seismic collapse shaking table test of BFMS conducted by the author’s research group^24^, a classical earthquake acceleration record for site II was selected. This record includes the Wolong wave from the 2008 Wenchuan Ms 8.0 earthquake, the Taiwan Jiji earthquake of 1999, and the El-Centro (Imperial Valley) earthquake of 1940. A three-directional (3D) ground motion was applied in accordance with the Code for Seismic Design of Buildings in China^21^. The Peak Ground Acceleration (PGA) ratios for the X, Y, and Z directions were set at 1: 0.85: 0.65. The seismic ground motion information of mainshocks is shown in Table 5.

**Table 5:** Seismic ground motion information of mainshocks.

Seismic ground motion name	Earthquake name	Magnitude	Time	Station name	Epicenter distance (Km)	Original horizontal PGA (g)	Adjusted PGA (g)	Interval (s)	Duration (s)
Wo-long	Wenchuan	8.0	2008	051WCW	24	0.958	0.62	0.005	75
ChiChi-TCU084	Jiji	7.6	1999	TCU084	13	1.009	0.62	0.005	75
ElCentro-BCJ	ElCentro	7.1	1940	BCJ	6	0.305	0.62	0.020	54

### Verification

**Fig. 9:**
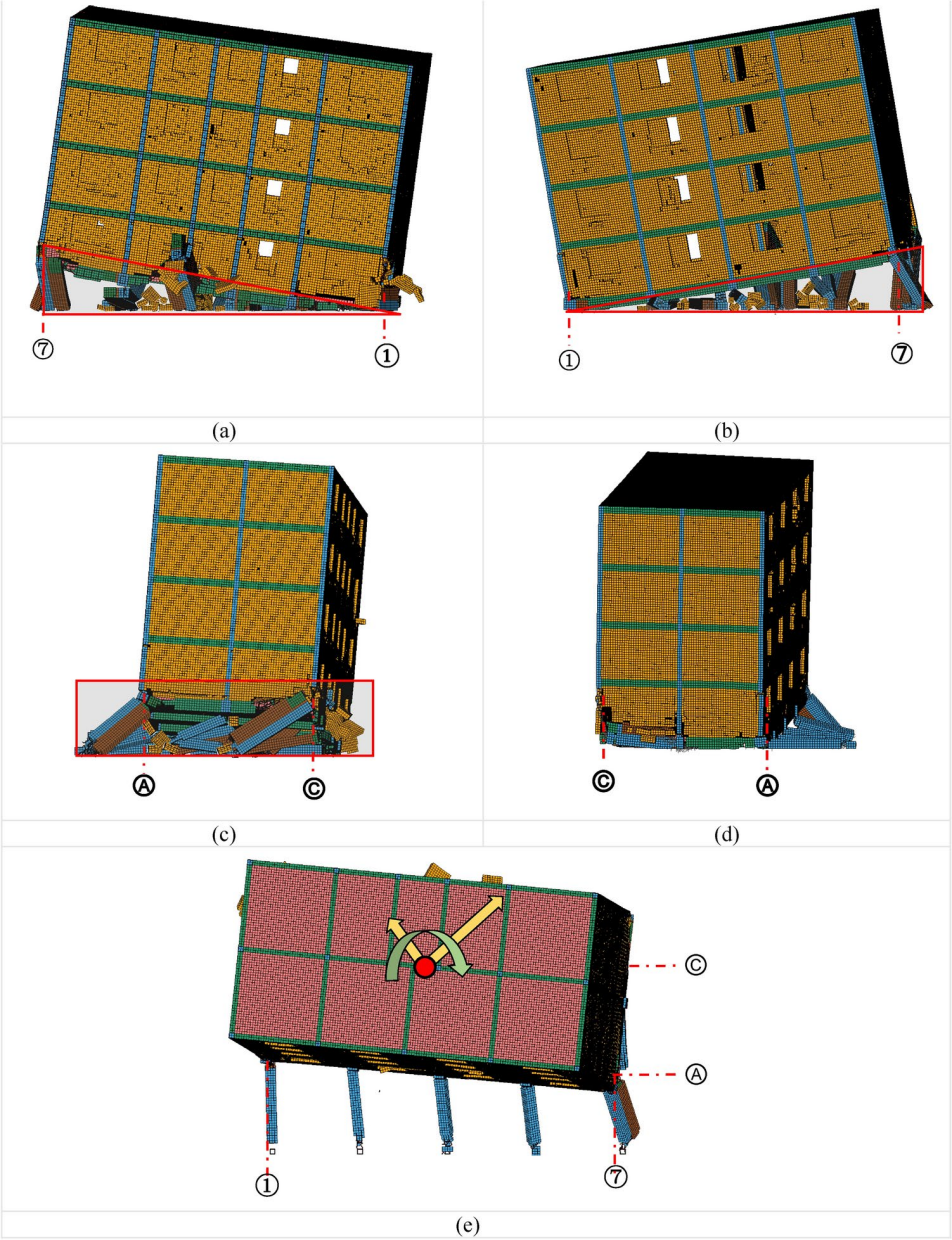
(**a**) ⑦-①-axle Collapse state of the BFMS. (**b**) ①-⑦-axle Collapse state. (**c**) Ⓐ-Ⓒ-axle Collapse stat. (**d**) Ⓒ**–**Ⓐ-axle Collapse state of the BFMS. (**e**) Top-view Collapse state.

**Fig. 10:**
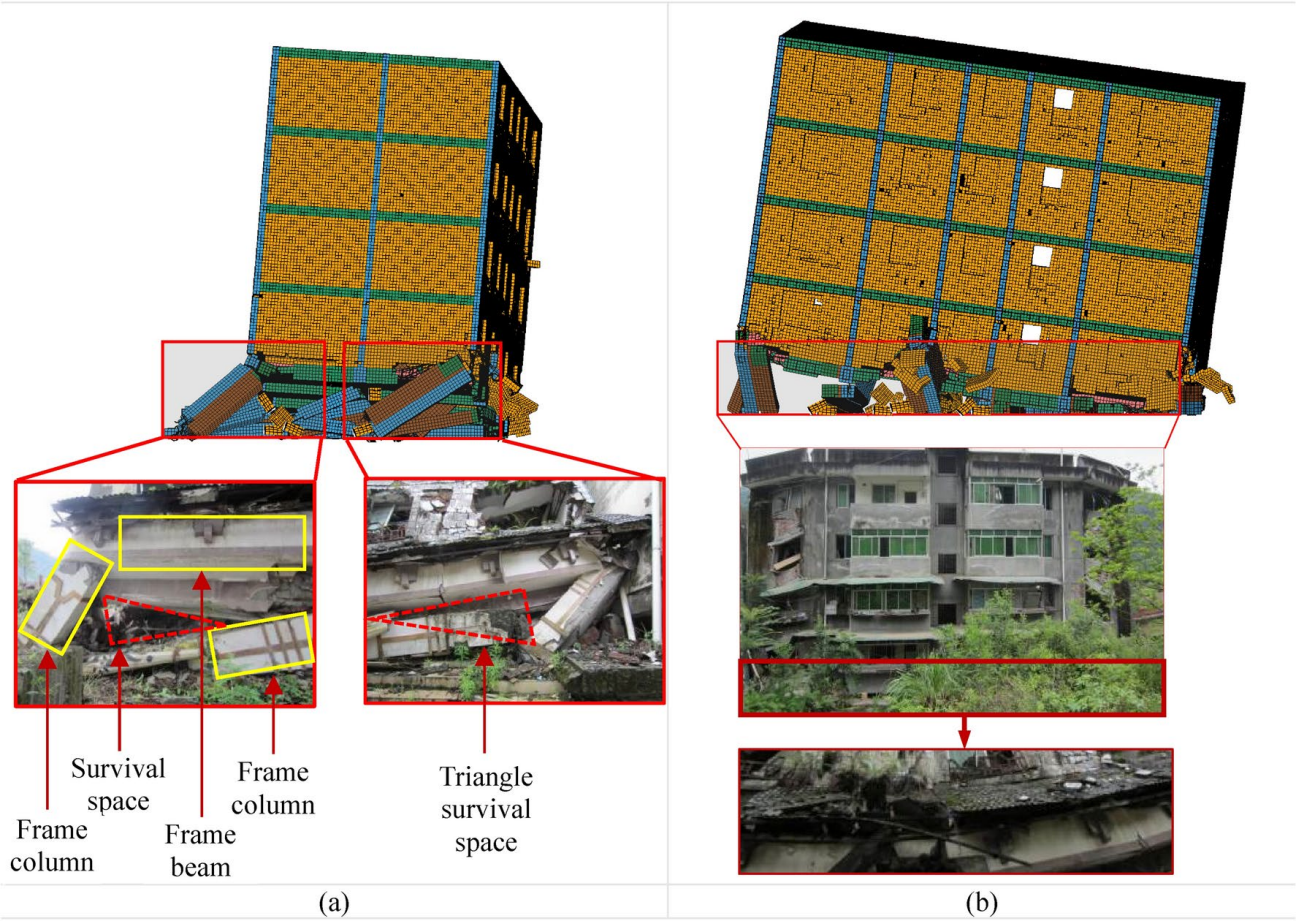
Thecomparison between the numerical model and actual seismic collapse of the BFMS: (**a**) BottomRCFstructure. (**b**) Front view.

**Fig. 11:**
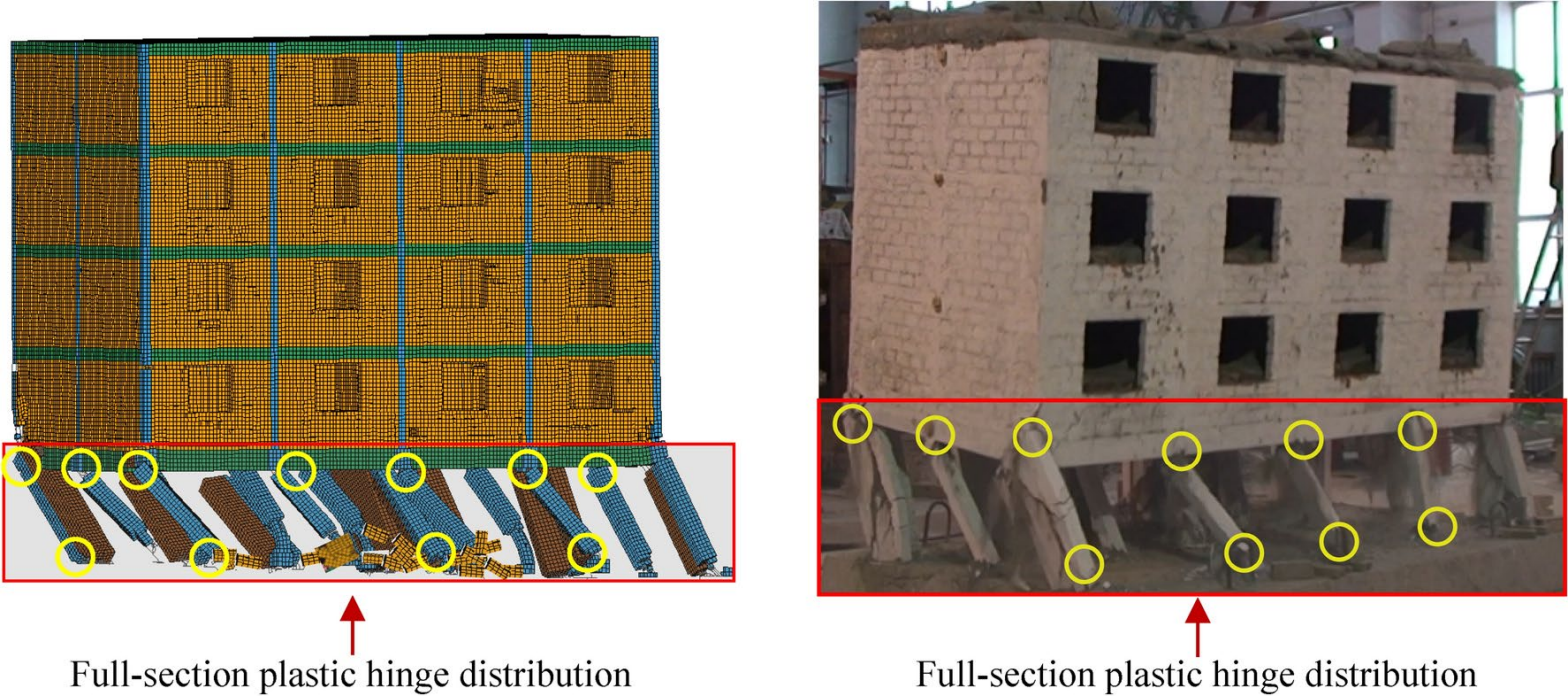
The comparison between the numerical model and shaking table test model.

1. Actual seismic damage verification
  Figure 9a–e illustrate the formation of BFMS-SR under the action of mainshocks. The BFMS-SR exhibits both half-seated and inclined collapse modes, which are consistent with the collapse modes observed in actual earthquake damage investigations^4^. The first-story RCF columns and seismic walls experienced semi-ruined collapse failures. The infilled walls underwent total collapse, with debris scattered between the ground and the first floor, seismic walls, and RCF beams. Notable damage is evident at the base of the two-story masonry structure, while the third to fifth stories remain largely intact, showing only a few diagonal cracks around the openings of masonry walls and windows. Overall, the BFMS exhibits significant twisting, as shown in Fig. 9e.
  Figure 10a–b compares the numerical model with the actual seismic collapse of BFMS-SR. In both instances, the bottom RCF structures form a triangular survival space between the RCF beam, the semi-collapsed bottom RCF column, and the seismic wall. Therefore, when using collapse mode as the evaluation criterion, the numerical simulation of the seismic collapse of the BFMS under mainshocks demonstrates a reasonable degree of reliability.
2. Shaking table test verification
  A comparison between the state of collapse in the numerical model and the shaking table test model (1/4-scale model)^24^ is presented, with identical loading cases applied to both models, as illustrated in Fig. 11. The comparison reveals that the damage patterns of the bottom RCF columns are similar, including the formation of full-section plastic hinges and significant tilting at the column ends. Additionally, the damage to the second to fifth layers appears minimal in both the numerical model and shaking table test model. However, due to the numerical model incorporating more layers than shaking table model, some structural columns and masonry walls in the second layer of the numerical model exhibit moderate damage under the same ground motion conditions.

## Mode and mechanism of secondary collapse for BFMS-SR

This section investigates the modes and mechanisms of secondary collapse in the BFMS-SR model under aftershocks, using the BFMS-SR model established from the mainshocks. It focuses on the principles for selecting aftershock sequences and introduces a simplified mechanical model for the BFMS-SR.

### Aftershock loading cases

This research conducted a statistical analysis of the spatial characteristics of aftershock sequences within the “72 h golden rescue” period following the 2008 Wenchuan earthquake, based on data from the China Earthquake Networks Center^34^. Additionally, spatial features of aftershock sequences from the 1975 Haicheng earthquake and the 1976 Longling earthquake were analyzed^35^. The conclusions drawn are as follows:

1. Aftershock sequences are characterized by their abundance, prolonged duration, and an overall trend of attenuation with fluctuations.
2. There is a high frequency of aftershocks in the early stages following the mainshock, with a gradual decrease in the number of aftershocks over time.
3. In the early stages of strong aftershocks, there is a notable absence of moderate-magnitude aftershocks. Figure 13 shows a significant decrease followed by an increase in seismic activity before strong aftershocks with magnitudes greater than 5.5, indicating a shortage of moderate aftershocks.
4. Aftershocks display alternating periods of high and low activity, demonstrating a quasi-periodic pattern.

Based on these spatial characteristics, two sets of 22 aftershocks, ranging from Ms 4.0 to Ms 6.1, from the 2008 Wenchuan aftershock sequences were selected to investigate the secondary collapse of the BFMS-SR model. A three-directional ground motion with a PGA ratio of X:Y: Z set at 1:0.85:0.65 was applied. Each aftershock was analyzed for an effective duration of 30 s, with the Small Restart Function in the LS-DYNA program used to implement the 11 aftershock cases in succession. Detailed information on aftershock cases 1 (AC-1) and 2 (AC-2) is provided in Tables 6 and 7.

**Table 6:** Aftershock cases 1 (AC1) information.

Aftershock cases	Ms	PGA (g)	Time (s)	Seismic station name	Seismic station place
AC1-1	6.0	0.331	30	051LXT080514122201	Taoping, Li County
AC1-2	4.2	0.055	30	051MXD080512162102	Diexi, mao County
AC1-3	4.7	0.078	30	051WCW080512163602	Wolong, Wenchuan
AC1-4	4.8	0.096	30	051LXT080512201303	Taoping, Li County
AC1-5	4.3	0.045	30	051WCW080512151303	Wolong, Wenchuan
AC1-6	5.2	0.188	30	051LXM080517042902	Miya, Li County
AC1-7	4.6	0.084	30	051LXT080512221001	Taoping, Li County
AC1-8	4.3	0.045	30	051WCW080512151303	Wolong, Wenchuan
AC1-9	4.5	0.065	30	051LXT080513012901	Taoping, Li County
AC1-10	5.6	0.239	30	051LXT080513074601	Taoping, Li County
AC2-11	4.4	0.058	30	051QCQ080512194102	Qiaolou, Qianchun County

**Table 7:** Aftershock cases1 (AC2) information.

Aftershock cases	Ms	PGA (g)	Time (s)	Seismic station name	Seismic station place
AC2-1	6.1	0.332	30	051AXY080514010102	Yong’an, An County
AC2-2	4.1	0.040	30	051WCW080512182302	Wolong, Wenchuan
AC2-3	4.6	0.084	30	051LXT080512221001	Taoping, Li County
AC2-4	4.9	0.098	30	051GYQ080527163701	Guangyuan, zhongyuan County
AC2-5	4.2	0.055	30	051MXD080512162102	Diexi, mao County
AC2-6	5.3	0.199	30	051LXT080513050801	Taoping, Li County
AC2-7	4.7	0.078	30	051WCW080512163602	Wolong, Wenchuan
AC2-8	4.2	0.055	30	051MXD080512162102	Diexi, mao County
AC2-9	4.6	0.084	30	051LXT080512221001	Taoping, Li County
AC2-10	5.7	0.221	30	051SMX080609152802	Xianfeng, Shimian County
AC2-11	4.4	0.058	30	051QCQ080512194102	Qiaolou, Qianchun County

### Measuring points

**Fig. 12:**
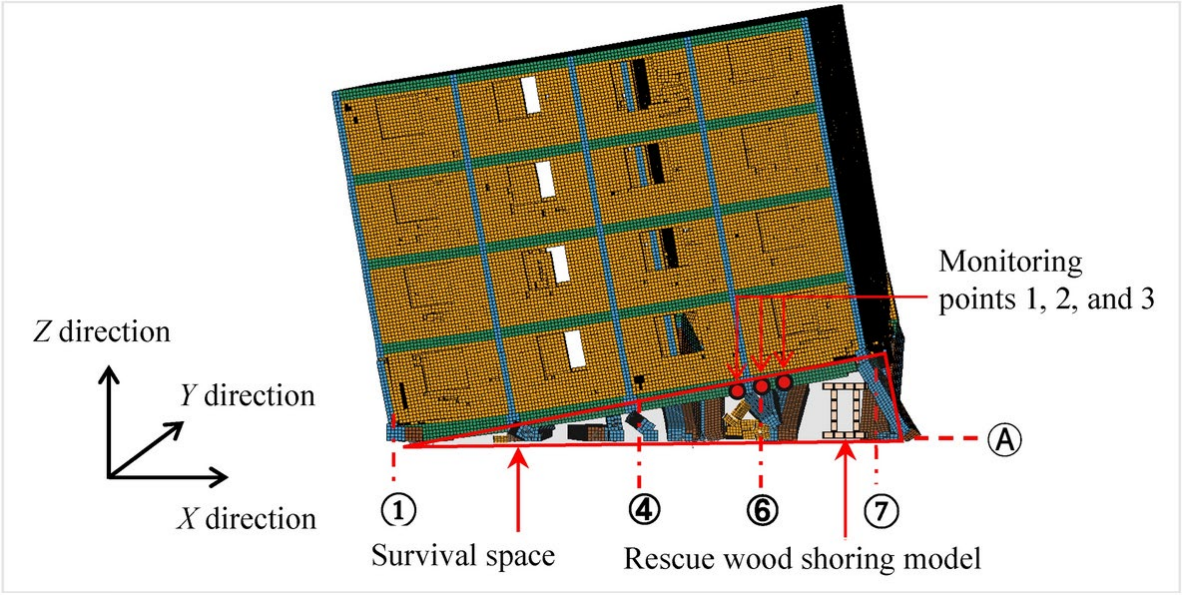
Monitoring points of BFMS-SR model.

To assess the structural dynamic response of the BFMS-SR model during secondary collapse under aftershocks, it is essential to select key monitoring points within the numerical model. Given that survival spaces exist at the bottom of the BFMS-SR model, monitoring these areas is critical, as changes can impact the safety of rescuers during post-earthquake operations. Considering the potential for both half-seated and inclined collapse modes in the BFMS-SR model under aftershocks, three key monitoring points were selected. These points are located at the intersections of the ⑥-axis and the Ⓐ-axis of the first-floor slab, as illustrated in Fig. 12.

### Secondary collapse mode

**Fig. 13:**
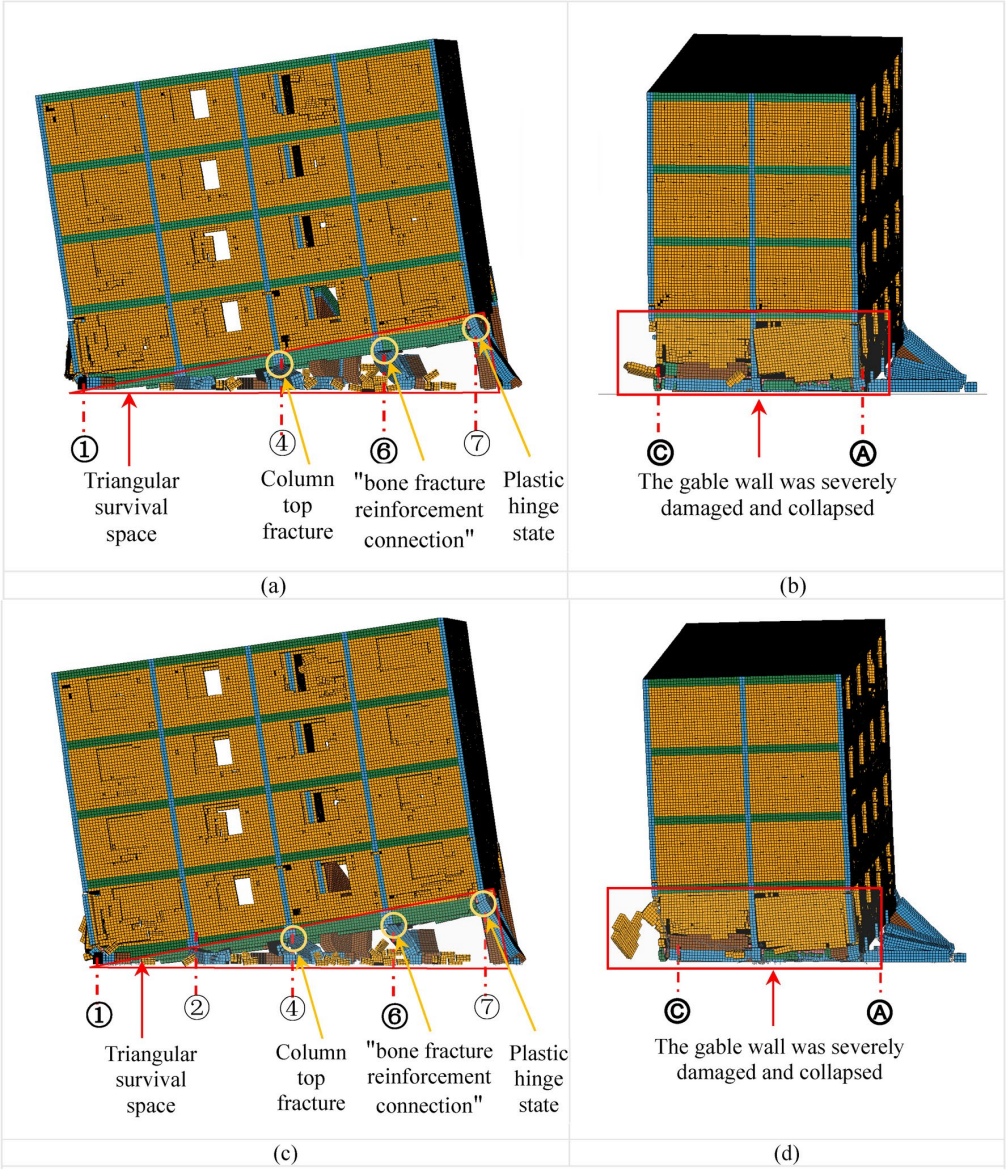
The secondary collapse process of BFMS-SR model: (**a**) ①-⑦-axle BFMS-SR model under the AC1-1. (**b**) Ⓒ-Ⓐ-axle BFMS-SR model under the AC1-1. (**c**) ①-⑦-axle BFMS-SR model under the AC2-1. (**d**) Ⓒ-Ⓐ-axle BFMS-SR model under the AC2-1.

**Fig. 14:**
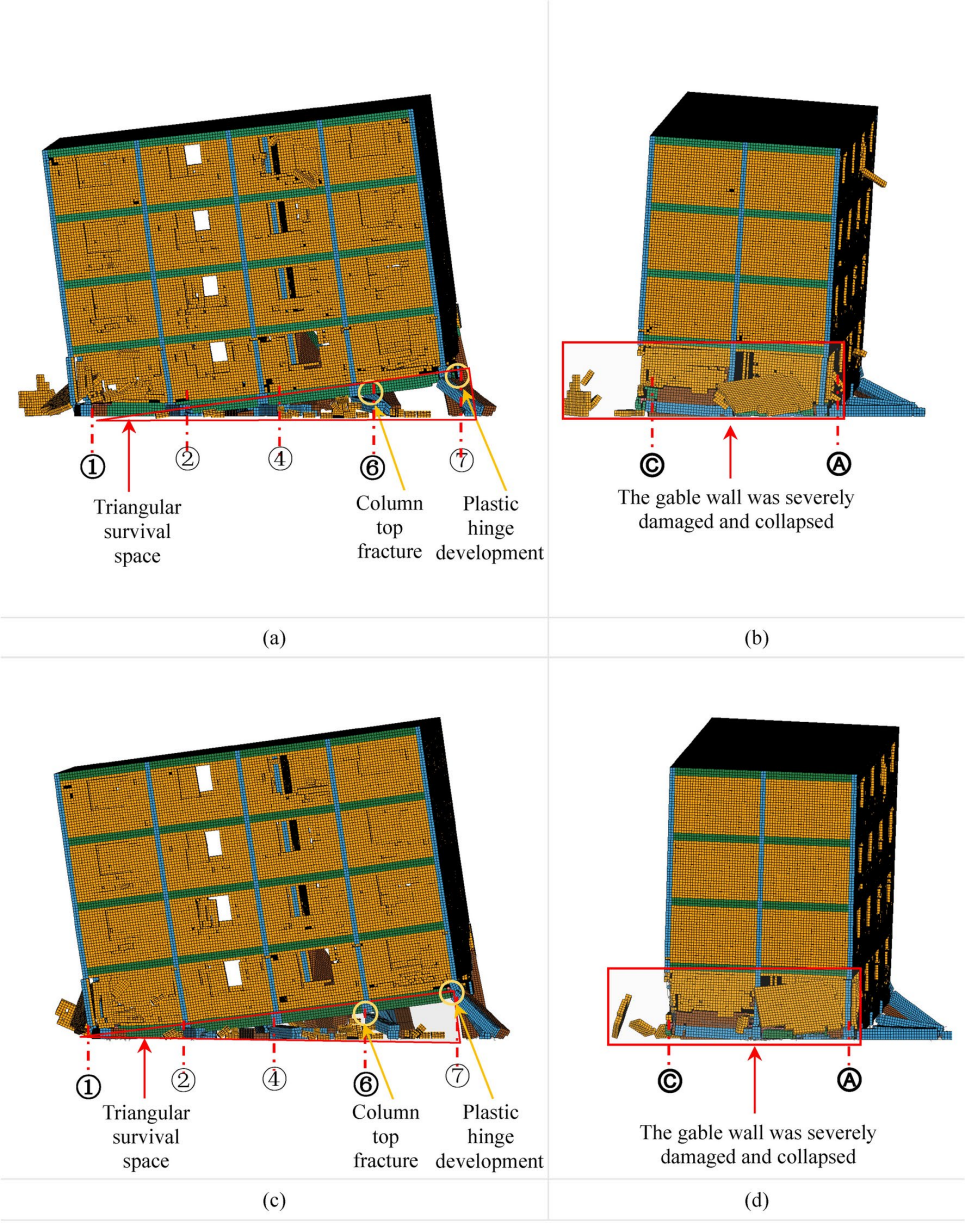
The secondary collapse process of BFMS-SR model: (**a**) ①-⑦-axle BFMS-SR model under the AC1-6. (**b**) Ⓒ-Ⓐ-axle BFMS-SR model under the AC1-6. (**c**) ①-⑦-axle BFMS-SR model under the AC2-5. (**d**) Ⓒ-Ⓐ-axle BFMS-SR model under the AC2-5.

**Fig. 15:**
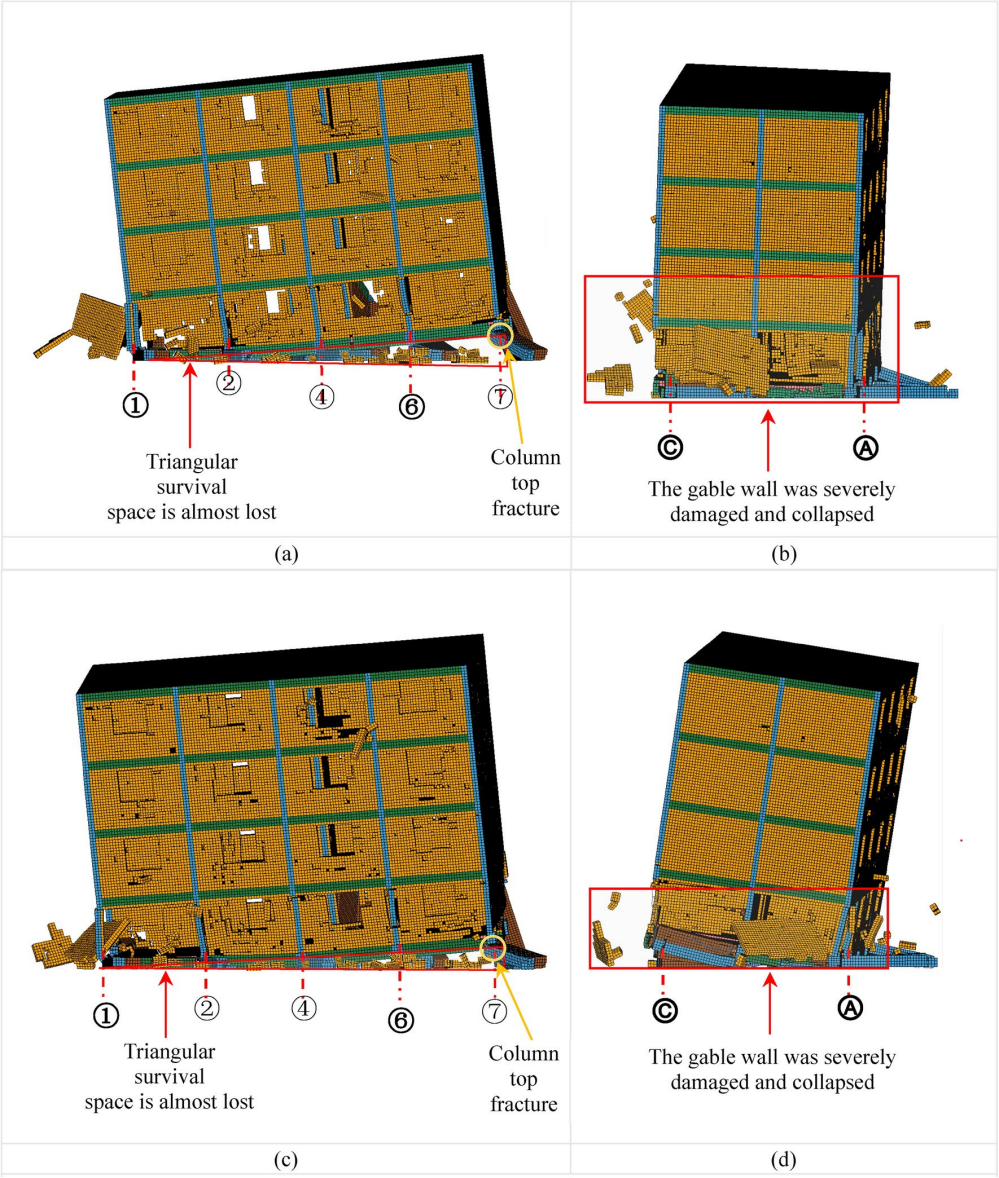
The secondary collapse process of BFMS-SR model: (**a**) ①-⑦-axle BFMS-SR model under the AC1-10. (**b**) Ⓒ-Ⓐ-axle BFMS-SR model under the AC1-10. (**c**) ①-⑦-axle BFMS-SR model under the AC2-9. (**d**) Ⓒ-Ⓐ-axle BFMS-SR model under the AC2-9.

Under the action of aftershocks AC1 and AC2, the vertical displacement of the BFMS-SR model progressively increases, leading to a gradual reduction in the surviving space area within the bottom layer. Eventually, the BFMS-SR model collapses into a fully seated-type secondary collapse under both AC1 and AC2. Significant vertical deformation is observed in the BFMS-SR model during the aftershocks AC1-1, AC1-6, AC1-10, AC2-1, AC2-5, and AC2-9, as illustrated in Figs. 13, 14, and 15. The main conclusions drawn from these observations are as follows:

*AC1-1 and AC2-1 Damage* The damage observed in the BFMS-SR model under AC1-1 and AC2-1 is similar. In both cases, the bottom RCF structure retains a distinct triangular survival space. At the end of the RCF column intersecting the ⑦-axis and the Ⓐ-axis, significant plastic deformation is evident, although some residual bearing capacity remains. At the end of the RCF column intersecting the ⑥-axis and the Ⓐ-axis, the concrete has severely peeled off, leaving the steel bars intact, which forms a “bone fracture reinforcement connection.” Conversely, the end of the RCF column at the ④-axis and Ⓐ-axis intersection has nearly lost its bearing capacity. Additionally, the gable wall on the second story at the ①-Axis has suffered severe damage, with portions of the masonry walls having fallen off, as depicted in Fig. 13a–d.

*AC1-6 and AC2-5 Damage* Under AC1-6 and AC2-5, the damage to the BFMS-SR model shows similar characteristics. The triangular survival space at the bottom RCF structure progressively diminishes. The plastic hinge at the end of the RCF column intersecting the ⑦-axis and the Ⓐ-axis continues to develop, leading to a further reduction in residual bearing capacity. At the end of the RCF column where the ⑥-axis intersects with the Ⓐ-axis, the concrete has severely peeled away, the steel bars have fractured, and the bearing capacity has been lost. The damage to the gable wall on the second story at the ①-Axis intensifies, causing severe collapse of the masonry walls, as shown in Fig. 14a–d.

*AC1-10 and AC2-9 Damage* The damage to the BFMS-SR model under AC1-10 and AC2-9 exhibits similar features. The triangular survival space in the bottom RCF structure is nearly entirely lost. The end of the RCF column at the intersection of the ⑦-axis and the Ⓐ-axis is severely damaged, resulting in the loss of remaining bearing capacity. The gable wall on the second story at the ①-Axis has collapsed and fallen off. Following 11 aftershocks, the BFMS-SR model ultimately transitions to a complete seated-type secondary collapse mode. The vertical load-bearing components in the bottom layer collapse entirely, leaving no survival space. At this point, the BFMS-SR model reaches a state of relative stability, as depicted in Fig. 15a–d.

Under the influence of two sets of aftershocks, the BFMS-SR model exhibits some variation in failure phenomena under identical aftershock conditions. Moreover, despite the similarities in the number and magnitude of aftershocks, the overall behavior of the BFMS-SR model consistently demonstrates a complete seated-type secondary collapse mode. This includes the total collapse of the bottom RCF columns and seismic wall. The upper masonry structure experiences a consistent level of damage across scenarios, with the overall performance remaining largely intact except for severe damage to some gable walls on the second story.

### Secondary collapse mechanism

**Fig. 16:**
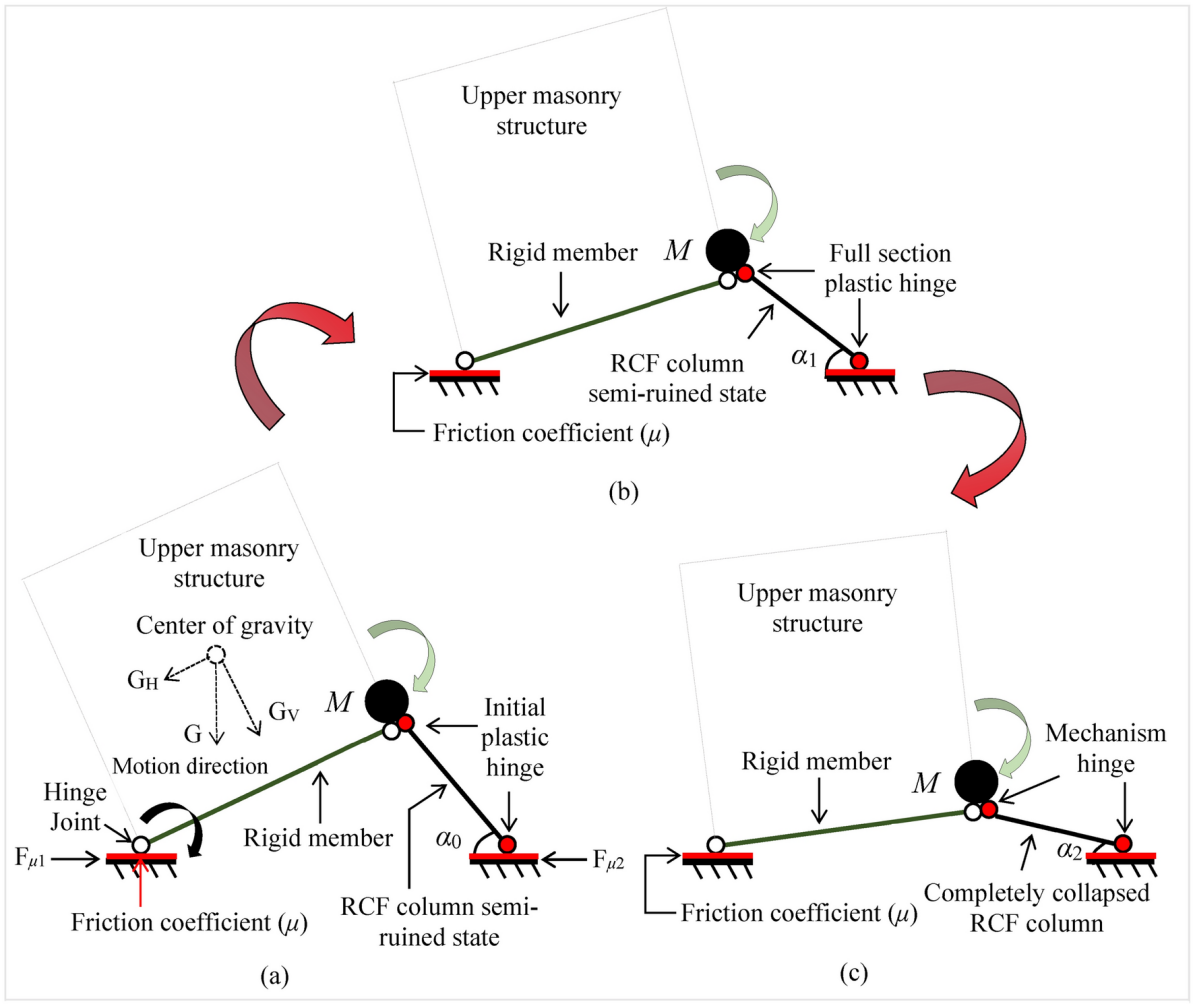
The calculation diagram of the BFMS-SR model.

The BFMS model undergoes a transformation into a semi-seated and inclined BFMS-SR model during the mainshocks. This is characterized by a triangular survival space within the bottom RCF structure. The transformation is primarily due to column hinge failures and a “strong beam weak column” mechanism, resulting in plastic hinges at the column ends. The bottom RCF structure then exhibits a “concrete peeling and steel yielding” mechanism, capable of withstanding bending moments, shear forces, and axial forces. The upper masonry structure remains nearly intact. Based on these damage features, a simplified calculation diagram of the BFMS-SR model is shown in Fig. 16. In this representation:

1. The bottom RCF structure is modeled with plastic hinges at the ends and an inclination angle (*α*_0_) of the RCF column.
2. The upper masonry structure is represented as a mass point (*M*) and a rigid member supporting this mass point.
3. The Connections between the ground and the bottom RCF column, as well as between the rigid member and the ground, are simplified as plastic hinge support and hinge support with sliding friction, respectively.

The BFMS-SR model can withstand a certain degree of aftershock loads, particularly during minor magnitude aftershocks, such as AC1-2, AC1-3, AC2-2, and AC2-3. During these aftershocks, the BFMS-SR model does not experience significant vertical deformation and retains a certain level of load-bearing capacity. Consequently, it poses a relatively minor threat to the safety of rescuers during post-earthquake rescue operations. The primary reason is that the plastic hinges in the RCF column are in their initial stage and can bear specific bending moments and shear forces, thereby effectively dissipating energy during aftershocks. At this stage, the RCF column is in a semi-ruined state: the concrete in the core area at the end of the RCF column is not crushed, and the reinforcement bars are yielding (see Fig. 16a).

However, as the magnitude of the aftershock increases, the BFMS-SR model undergoes secondary collapse. The primary reason is that the energy dissipation capacity of the RCF column’s plastic hinges is less than the energy input from the aftershocks to the BFMS-SR model. At this stage, the RCF column remains in a semi-ruined state, and its inclination angle (*α*_1_) decreases. Most of the concrete in the core area of the RCF column is crushed, and some reinforcement bars fracture. Finally, the plastic hinges of the RCF column have evolved into a full-section plastic hinge state (see Fig. 16b).

Nevertheless, the BFMS-SR model retains some load-bearing capacity due to entering a new equilibrium state. As aftershocks continue to impart energy, the BFMS-SR model undergoes secondary collapse once more. At this stage, the RCF column is in a completely collapsed state, and its inclination angle (*α*_2_) becomes minimal. The concrete in the core area of the RCF column is completely crushed, and the reinforcement bars are completely fractured. Finally, the plastic hinges of the RCF column have evolved into a mechanism hinge state (see Fig. 16c).

Due to the influence of building ruins, such as infill walls, the BFMS-SR model does not achieve a complete seated-type ruin state, and the inclination angle (*α*_2_) of the RCF column does not decrease to zero (see Fig. 15c,d). The formation of survival spaces and the construction of rescue passages in the BFMS-SR are both influenced by the ruins of infill walls. Therefore, it is essential to account for nonstructural components, such as infill walls, when simulating the seismic collapse of the BFMS. The stability of the BFMS-SR is compromised by the frictional force (F_*μ*1_) and the plastic bearing capacity of the RCF column end under gravitational load (G). The BFMS-SR tilts towards the direction of the frictional force (F_*μ*1_) and maintains direct contact with the ground. Concurrently, the bearing capacity of the RCF column end gradually diminishes due to the effects of aftershocks, resulting in a progressive collapse of the BFMS-SR in the direction of the RCF column. As fractures develop at the RCF column end, it becomes apparent that F_*μ*1_ greatly exceeds F_*μ*2_, ultimately leading to the collapse state depicted in Fig. 16c. In summary, the secondary collapse mechanism of the BFMS-SR is characterized by the “column hinge development failure mechanism,” which offers valuable insights for the rapid assessment of earthquake rescue sites^7^ and the optimal placement of rescue wood shoring.

## Main characteristics of secondary collapse of BFMS-SR model

### Secondary collapse discrimination

**Fig. 17:**
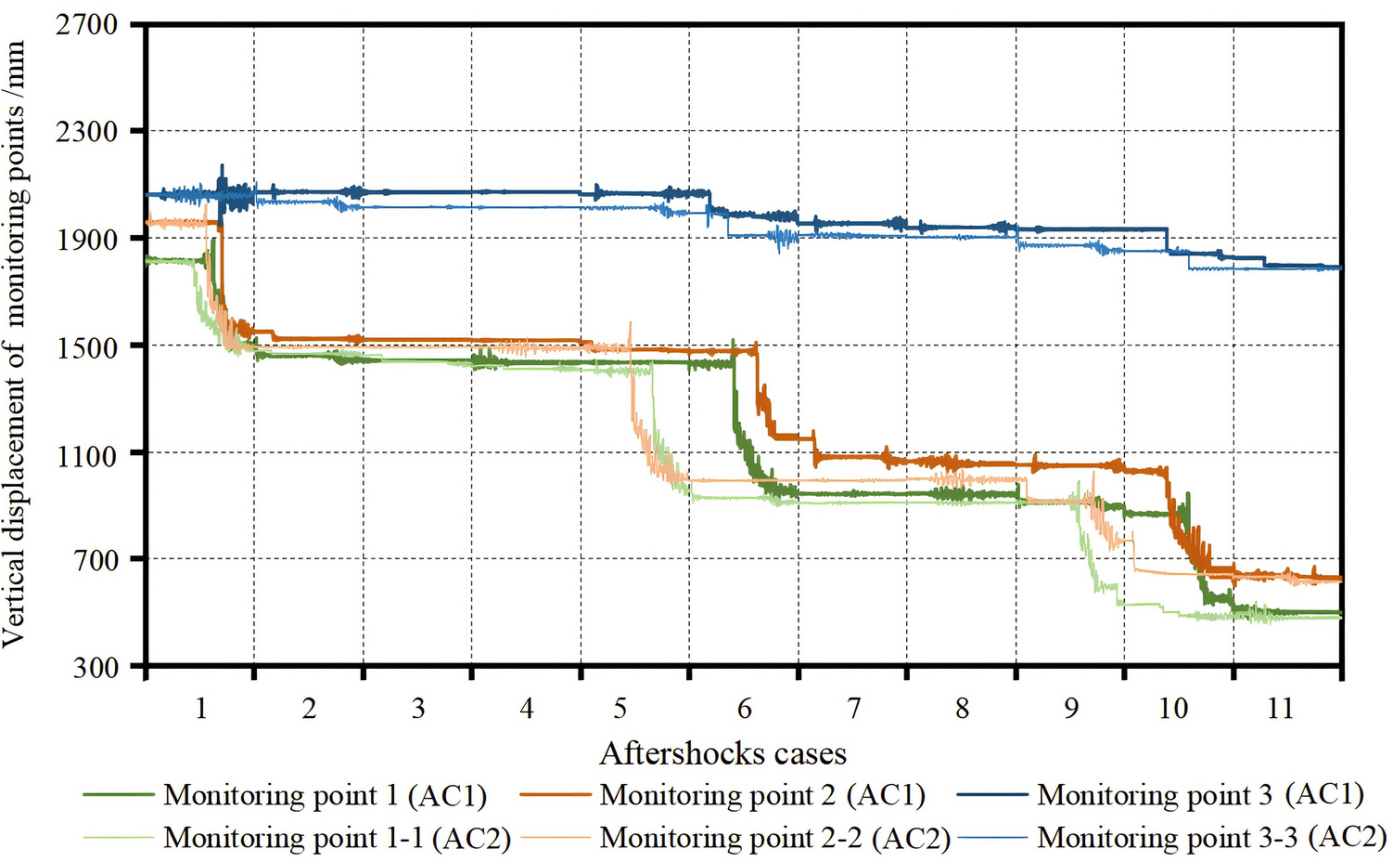
The vertical displacement time-history curve of points 1, 2, 3 of BFMS-SR under AC1 and AC2.

The vertical displacement time-history curves for measurement points 1, 2, and 3 of the BFMS-SR model under the influence of AC1 and AC2 are shown in Fig. 17. Specifically, the vertical displacement at measurement points 1 and 2 of the BFMS-SR model undergoes significant changes during AC1-1, AC1-6, AC1-10, and AC2-1, AC2-5, and AC2-9. Conversely, the vertical displacement at measurement point 3 of the BFMS-SR model remains insignificant during both AC1 and AC2. The vertical displacement values at measurement points 1 and 2 during AC1 and AC2 are detailed in Table 8.

**Table 8:** The vertical displacements of measurement points 1 and 2 under AC1 and AC2.

Aftershock cases	AC1-1 (mm)	AC1-6 (mm)	AC1-10 (mm)	AC2-1 (mm)	AC2-5 (mm)	AC2-9 (mm)
The vertical displacement of point 1	376	641	408	398	634	413
The vertical displacement of point 2	398	375	415	425	575	329

The occurrence of secondary collapse in building ruins is primarily evaluated based on whether it poses a threat to the safety of rescuers and trapped individuals, considering earthquake rescue operations. A key indicator of secondary collapse is the significant vertical displacement of building ruins during aftershocks. Secondary collapse is considered to have occurred when the vertical displacement of building ruins exceeds a certain threshold. In earthquake rescue operations, wood shoring plays a crucial role in constructing life-saving passages within building ruins^36^. DeFeng Xu^8^ conducted bearing capacity tests on rescue wood shoring in 2021, establishing a relationship between varying heights, vertical bearing capacity, and displacement. Building ruins are considered to have undergone secondary collapse during aftershocks when the yield displacement of the wood shoring is exceeded. Selecting appropriate rescue shoring is crucial for the safe construction of life-saving passages. The load-bearing capacity (*F*) of rescue shoring is determined by various factors, including the bearing area (*S*), the number of floors above the ruins (*N*), the design static load of materials (*P*), and the bearing capacity of the vertical structural components of the building ruins (*R*). These relationships are expressed in Eqs. (3) and (4).

3 $$F \leqslant F_{d}$$

4 $$F = N \times S \times P \times ({1} - R)$$

where *F*_*d*_ represents the design value of the rescue wood shoring, the *P* value is obtained from the “Guidelines for Urban Search and Rescue Shoring Operations”^37^, and the *R* value is obtained from the “Criteria for damage assessment of reinforced concrete and steel reinforced concrete buildings’^38^.

Based on the characteristics of the BFMS-SR model and the installation location of the rescue wood shoring at the intersections of the ⑥ and ⑦-axis with the Ⓐ-axis (in Fig. 12), the minimum required value for *F*_*d*_ is determined to be ≥ 2750 kN, as calculated from Eq. (4). The vertical displacements during the secondary collapse of the BFMS-SR model are 267 mm, 295 mm, and 324 mm, respectively, as documented by DeFeng Xu^39^. It is observed that the BFMS-SR model experienced secondary collapse during AC1-1, AC1-6, AC1-10, AC2-1, AC2-5, and AC2-9, as confirmed by comparing with Table 8.

### Main characteristics

**Fig. 18:**
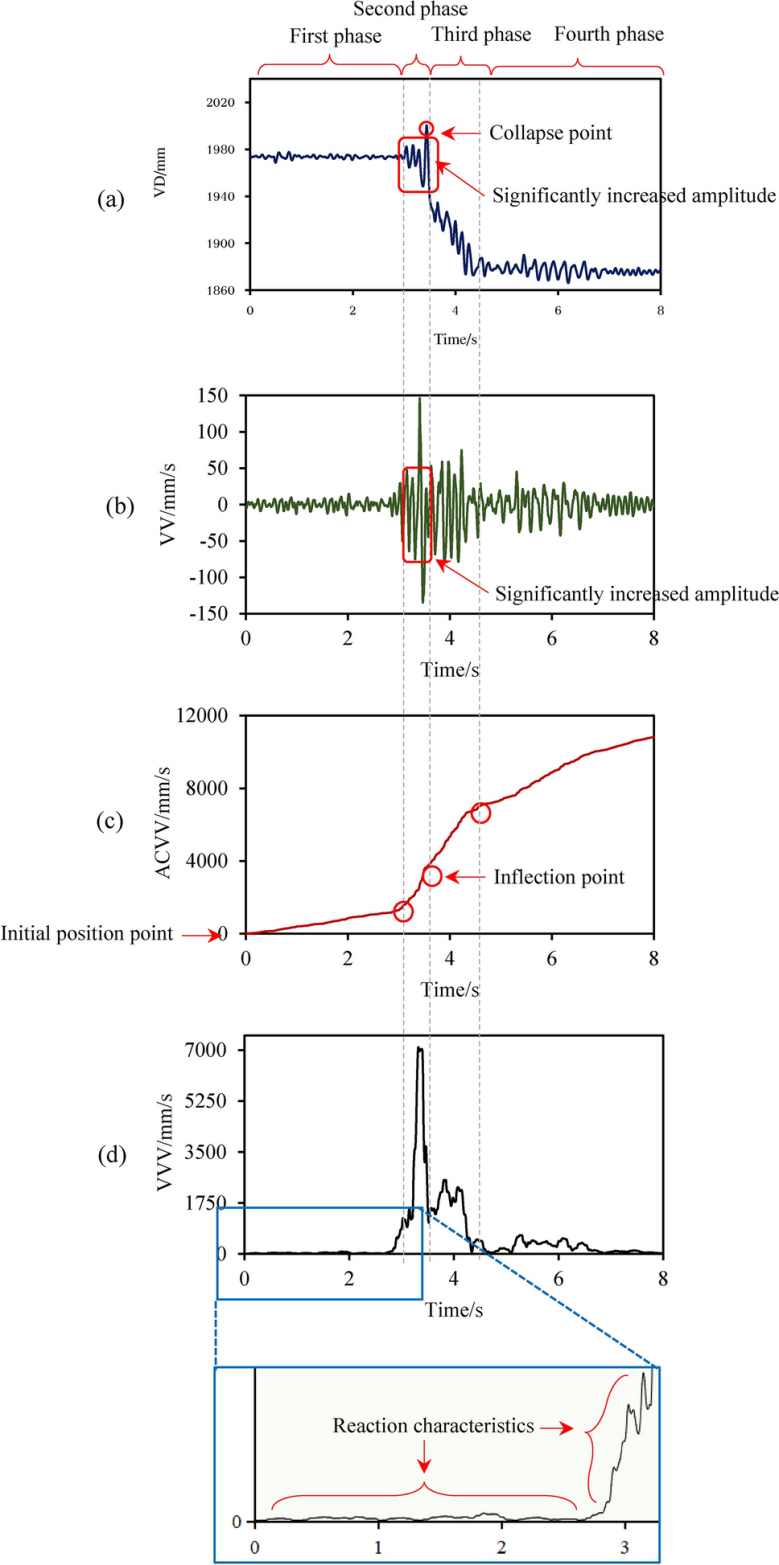
The variation characteristics of time-history curve of VD, VV, ACVV, and VVV.

Using measurement point 1 under AC1, the vertical displacement (VD) and vertical velocity (VV) time-history curves are shown in Fig. 18a,b. Additionally, the absolute value accumulation of vertical velocity (ACVV) time-history curve is shown in Fig. 18c. The variance of the vertical velocity (VVV) time-history curve is calculated at intervals of every 20 time points, as depicted in Fig. 18d.

1. Figure 18a,b display a significant increase in both VD and VV amplitudes before the secondary collapse of the BFMS-SR model. The vertical displacement fluctuates around its initial value, without significant residual downward displacement. This phenomenon indicates the development of a plastic hinge at the column end of the BFMS-SR model, leading to a decrease in overall stiffness and the accumulation and intensification of damage.
2. Figure 18c shows that the ACVV time-history curve exhibits distinct inflection points. The ACVV time-history curve of the secondary collapse of the BFMS-SR model reveals four stages: stabilization (T1), development of structural response (T2), secondary collapse (T3), and post-collapse (T4).
3. The ACVV time-history curve reflects the damage accumulation in the BFMS-SR model under aftershocks. This curve can be interpreted as the displacement of the BFMS-SR model from its initial position, showing gradual increases over time (Fig. 18c). The trend of the ACVV time-history curve reflects the evolving risk of secondary collapse in the BFMS-SR model under aftershocks.
4. Figure 18d reveals that the VVV time-history curve reflects the stability of the BFMS-SR model under aftershocks. Similarly, the variance of the vertical displacement (VVD) time-history curve exhibits similar characteristics. Consequently, both VVV and VVD can characterize the primary features related to the secondary collapse of the BFMS-SR model under aftershocks.

**Fig. 19:**
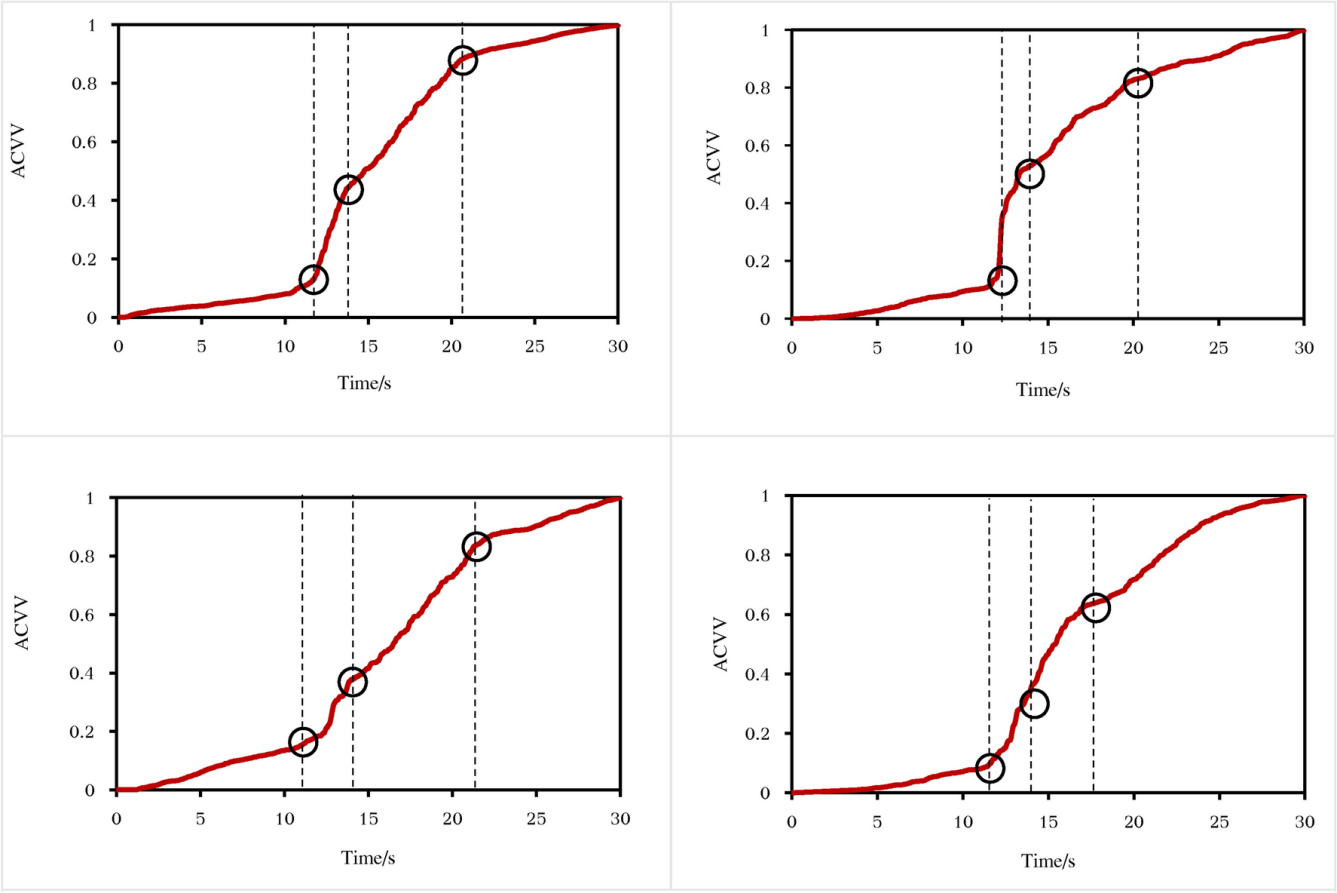
Accumulated time-history of absolute vertical velocity (ACVV) (Measuring point 1 and 2).

**Fig. 20:**
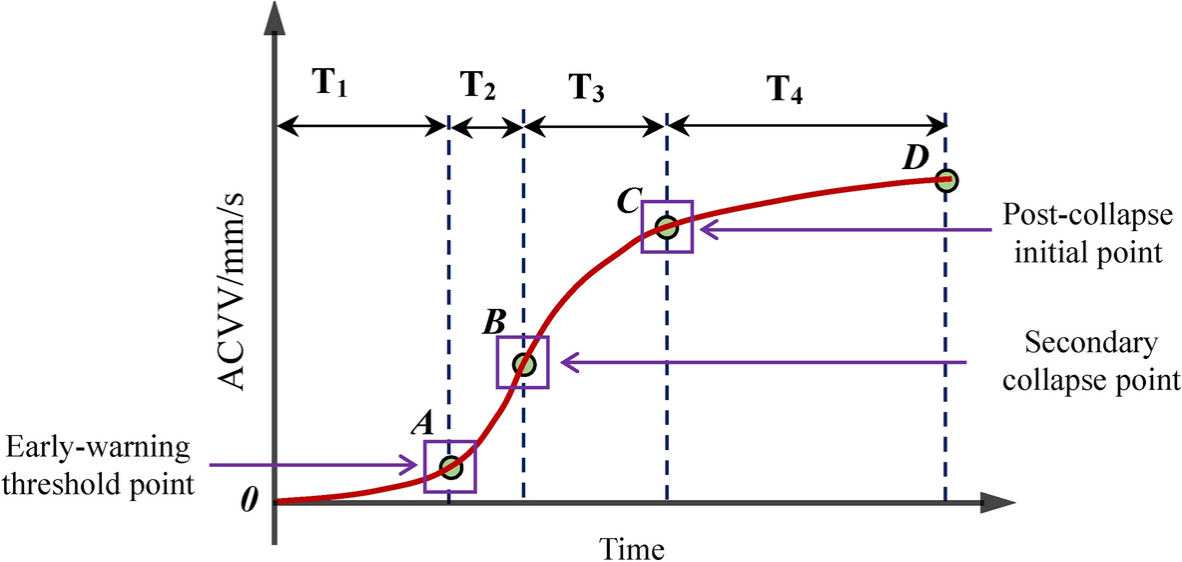
The variety rule of accumulated time-history of absolute vertical velocity (ACVV).

The ACVV time-history curves of monitoring points 1 and 2 on the BFMS-SR model under the influence of AC1-1 and AC2-5 exhibit the same four stages as previously observed, confirming their universality (see Fig. 19). The variation pattern of the ACVV time-history curves of the BFMS-SR model is illustrated in Fig. 20. The key characteristics of the four stages are as follows:

1. **Stabilization stage (T1):** This stage is characterized by a slow, linear increase in the slope of the ACVV time-history curve and a gradual rise in the VD time-history curve. During this period, the BFMS-SR model retains a certain degree of stability. The bottom RCF structure of the BFMS-SR model maintains a stable triangular survival space.
2. **Structural response development stage (T2)**: During this stage, the slope of the ACVV time-history curve increases rapidly, accompanied by a swift rise in the VD time-history curve. Amplitude points fluctuate around their initial values, indicating that the plastic hinge at the end of the RC frame column, which has not yet collapsed, continues to develop. The stiffness of the bottom structure begins to decrease, and the accumulation of damage intensifies. At this stage, the RC frame columns that have not collapsed still retain some bearing capacity (as shown in the column at the intersection of the ⑦-axis and the Ⓐ-axis in Fig. 12). Signs of sudden changes in the structural dynamic response of the BFMS-SR model are evident, with vertical velocity values decreasing from their maximum to zero.
3. **Secondary collapse stage (T3):** This stage is characterized by a sharp increase in the slope of the ACVV time-history curve, followed by a period of linear development. The vertical displacement begins to decrease rapidly, indicating that most of the frame columns have lost their bearing capacity.
4. **Post-collapse stage (T4):** During this stage, the amplitude of the VD time-history curve decreases gradually, trending towards stability. This indicates that the BFMS-SR model has reached a new equilibrium state.

The derived ACVV variety rule for the secondary collapse of the BFMS-SR model under aftershocks provides a valuable foundation for developing earthquake rescue early-warning systems. Monitoring the structural dynamic response of the BFMS-SR model during aftershocks allows for the establishment of an early-warning threshold. When the monitored response values reach this threshold, an automatic alarm is triggered, promptly notifying rescuers to evacuate the BFMS-SR, thereby ensuring their safety. As shown in Fig. 20, the slope of the curve at point A, marking the junction of the T1 and T2 stages, increases significantly, exhibiting a noticeable turning point and rapid rise. The slope at point B, occurring at the end of the T2 stage, reaches its maximum value before gradually decreasing as the BFMS-SR model stabilizes. Therefore, the structural dynamic response value at point A, marking the start of the T2 stage, can be used as the early-warning threshold for the secondary collapse of the BFMS-SR model under aftershocks. Constructing earthquake rescue response operations based on this threshold allows for timely evacuation and the implementation of safety measures when the BFMS-SR model approaches the risk of secondary collapse. This approach enhances the safety of rescuers during earthquake rescue operations. The point at the end of the stabilization phase is also suggested as a potential early-warning threshold for the secondary collapse of the BFMS-SR model under aftershocks.

## Conclusion

We propose a numerical modeling framework for BFMS under main-aftershock conditions, utilizing the Finite Element Method-Finite Discrete Element Method (FEM–FDEM) in the LS-DYNA program. This study explores the methodology for selecting and inputting aftershocks into BFMS-RS. It investigates the incorporation of aftershocks into BFMS-RS and examines the secondary collapse modes and mechanisms under various aftershock scenarios. Additionally, it analyzes the structural dynamic response characteristics of BFMS-RS under diverse aftershock sequences. The following conclusions were drawn:

1. The numerical simulation framework, FEM-DFEM, accurately replicated the transition of BFMS from an intact state to semi-ruins and eventual secondary collapse under mainshock-aftershock conditions. The inclined semi-ruined state formed by the BFMS under mainshocks, and the seated collapse mode formed by BFMS-RS under aftershocks, are consistent with actual earthquake damage observations and shaking table test results.
2. Aftershock sequences are characterized by their abundance, prolonged duration, varying magnitudes, and typical periodicity, with an overall trend of attenuating fluctuations. These characteristics provide fundamental principles for incorporating aftershock cases into building ruins (including BFMS-RS), facilitating the investigation of secondary collapse phenomena.
3. The secondary collapse of BFMS-RS under aftershocks primarily follows a “column hinge development failure mechanism.” This mechanism offers valuable insights for optimizing the placement of rescue shoring during earthquake rescue operations.
4. Under the influence of two different sets of aftershock cases, the failure phenomenon of the BFMS-SR model under the same number of aftershock conditions is not entirely identical. Additionally, under the same number and magnitude of aftershock cases, the overall behavior of the BFMS-SR model consistently demonstrates a seated collapse mode, with nearly identical vertical displacement variations.
5. The BFMS-SR model exhibits a certain degree of stability; however, its vulnerability to secondary collapse escalates with the accumulation of structural damage, particularly under the impact of strong aftershocks. The absolute value accumulation of the vertical velocity (AAVV) time-history curve during the secondary collapse of BFMS-SR identifies four distinct stages: stabilization, structural response development, secondary collapse, and post-collapse. Additionally, the point marking the end of the stabilization phase is proposed as a potential early-warning threshold for the secondary collapse of the BFMS-SR model under aftershocks.

## Data Availability

The datasets used and/or analysed during the current study available from the corresponding author (email: hxzhao2009@163.com) or first auther (email: xudefeng_2008@126.com) on reasonable request.
